# Molecular docking of substituted pteridinones and pyrimidines to the ATP-binding site of the N-terminal domain of RSK2 and associated MM/GBSA and molecular field datasets

**DOI:** 10.1016/j.dib.2020.105347

**Published:** 2020-02-28

**Authors:** Kimberly A. Casalvieri, Christopher J. Matheson, Donald S. Backos, Philip Reigan

**Affiliations:** Department of Pharmaceutical Sciences, Skaggs School of Pharmacy and Pharmaceutical Sciences, University of Colorado Anschutz Medical Campus, 12850 East Montview Boulevard, Aurora, CO, 80045, USA

**Keywords:** RSK2, Kinase, Inhibitor, Structure-activity relationship, Molecular docking, QSAR

## Abstract

The data have been obtained for a series of substituted pteridinones and pyrimidines that were developed based on BI-D1870 to establish a structure-activity relationship for RSK inhibition. The 19 compounds, 12 of these with R- and S-isomeric forms, were docked into the ATP-binding site of the N-terminal domain of the RSK2 kinase using Schrodinger Glide. The binding conformations of these molecules and their interactions with RSK2 may inform the development of further small molecule RSK inhibitors. The molecular mechanics energies combined with the generalized Born and surface area continuum solvation (MM-BGSA) method was used to estimate the free energy of binding of the small molecules with RSK2. The molecular field characteristics of the docked confirmations of the inhibitors was examined using Cresset Forge software. The synthesis and evaluation of these compounds was reported in the related research article: Substituted pteridinones as p90 ribosomal S6 protein kinase 2 (RSK2) inhibitors: a structure-activity study (Casalvieri et al., 2020).

**Table:** Specifications Table

Subject	Drug Discovery
Specific subject area	Computational-based molecular docking and three-dimensional quantitative structure-activity relationship
Type of data	Tables, images, graphs, and figures
How data were acquired	PerkinElmer ChemDraw Prime, Schrodinger 2018-4 Glide and Prime, Cresset Forge
Data format	Raw, analyzed, and filtered
Parameters for data collection	The docking of the pteridinones and pyrimidines was targeted at a 6 Å radius area that encompassed the ATP-binding site of the N-terminal domain of RSK2 (PDB: 5D9K) using Glide.
Description of data collection	The MM/GBSA calculations were performed using Prime to estimate binding affinity of the pteridinones and pyrimidines to the binding site was performed using the VSGB solvation model. Then molecular field characteristics for each compound was determined using Forge.
Data source location	Institution: University of Colorado City/Town/Region: Aurora, Colorado 80045 Country: USA Latitude: 39° 44′ 25.41″ N; Longitude: 104° 50′ 9.47″ W
Data accessibility	Data is with this article.
Related research article	K. A. Casalvieri, C. J. Matheson, D. S. Backos, P. Reigan. Substituted pteridinones as p90 ribosomal S6 protein kinase 2 (RSK2) inhibitors: a structure-activity study. Bioorganic and Medicinal Chemistry, 2020, 28, (5), 115303.

**Table:** 

**Value of the Data** • The RSK2 kinase has been identified as a molecular target for the treatment of various cancer types. • The pteridinones and pyrimidines comprised a structure-activity study for BI-D1870, a potent pan-RSK inhibitor. • The modeling data was generated to guide the structure-activity study and to rationalize the structural requirements for RSK inhibition. • The binding confirmations of the pteridinones and pyrimidines, their interactions with RSK2 and calculated binding energies may inform further studies focused on the development of RSK inhibitors. • The molecular field models for the RSK inhibitors in their docked conformations provides additional information in terms of favourable electronics for RSK inhibitor binding.

## 1 Data description

**Fig. 1:**
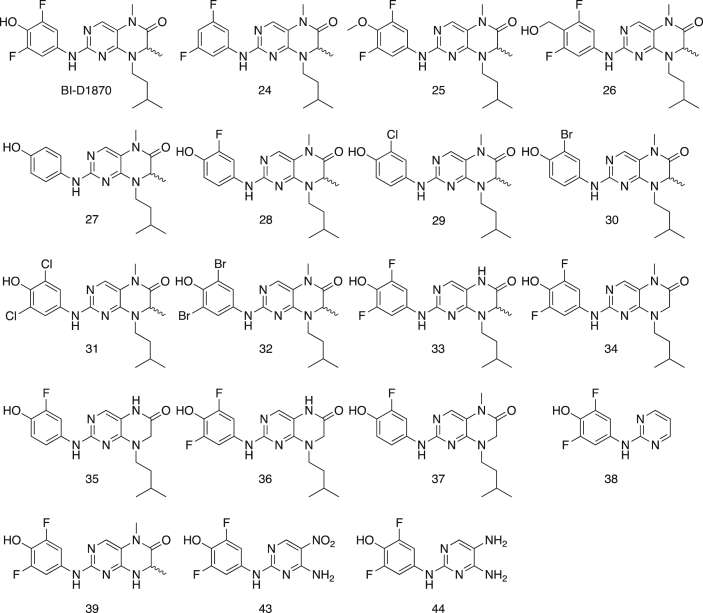
Chemical structures of substituted pyrimidines and pteridinones. Compound numbering retained from [11].

**Fig. 2:**
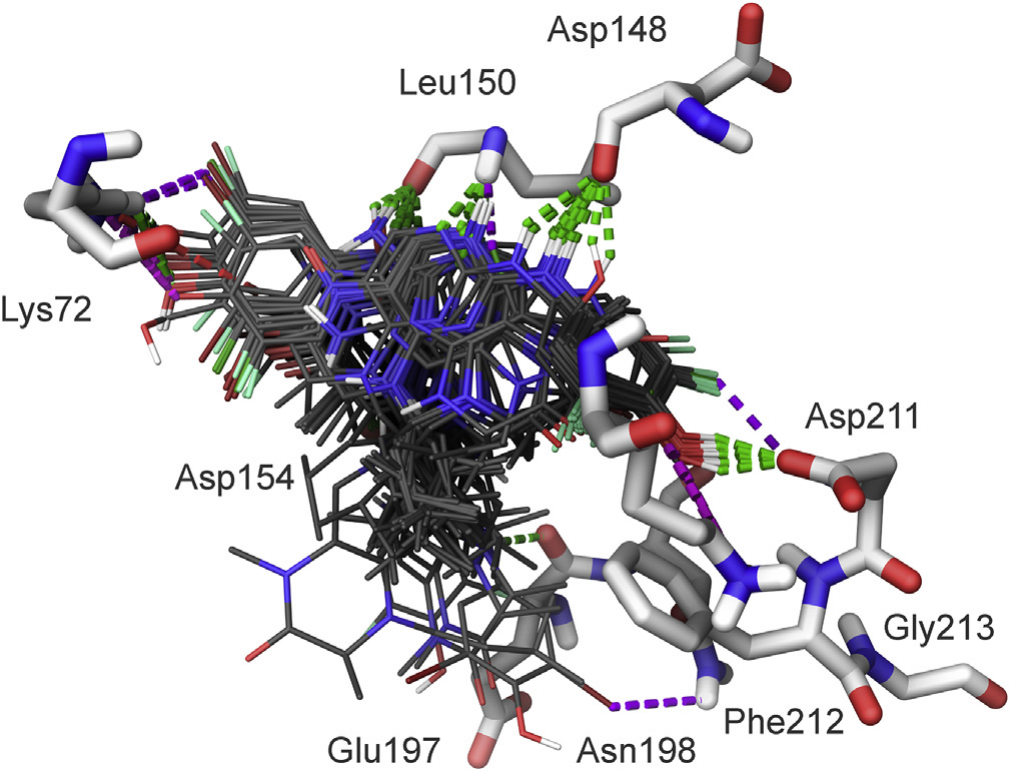
Stick display style representation of amino acid residues (carbons colored white) in the ATP-binding site of the NTKD of RSK2 and an overlay of docked conformations of the compounds (carbons colored black), where green dashed lines indicate H-bonds, violet dashed lines indicate halogen bonds, magenta dashed lines indicate salt bridges, and dark green dashed lines indicate Pi-cation interactions.

**Fig. 3:**
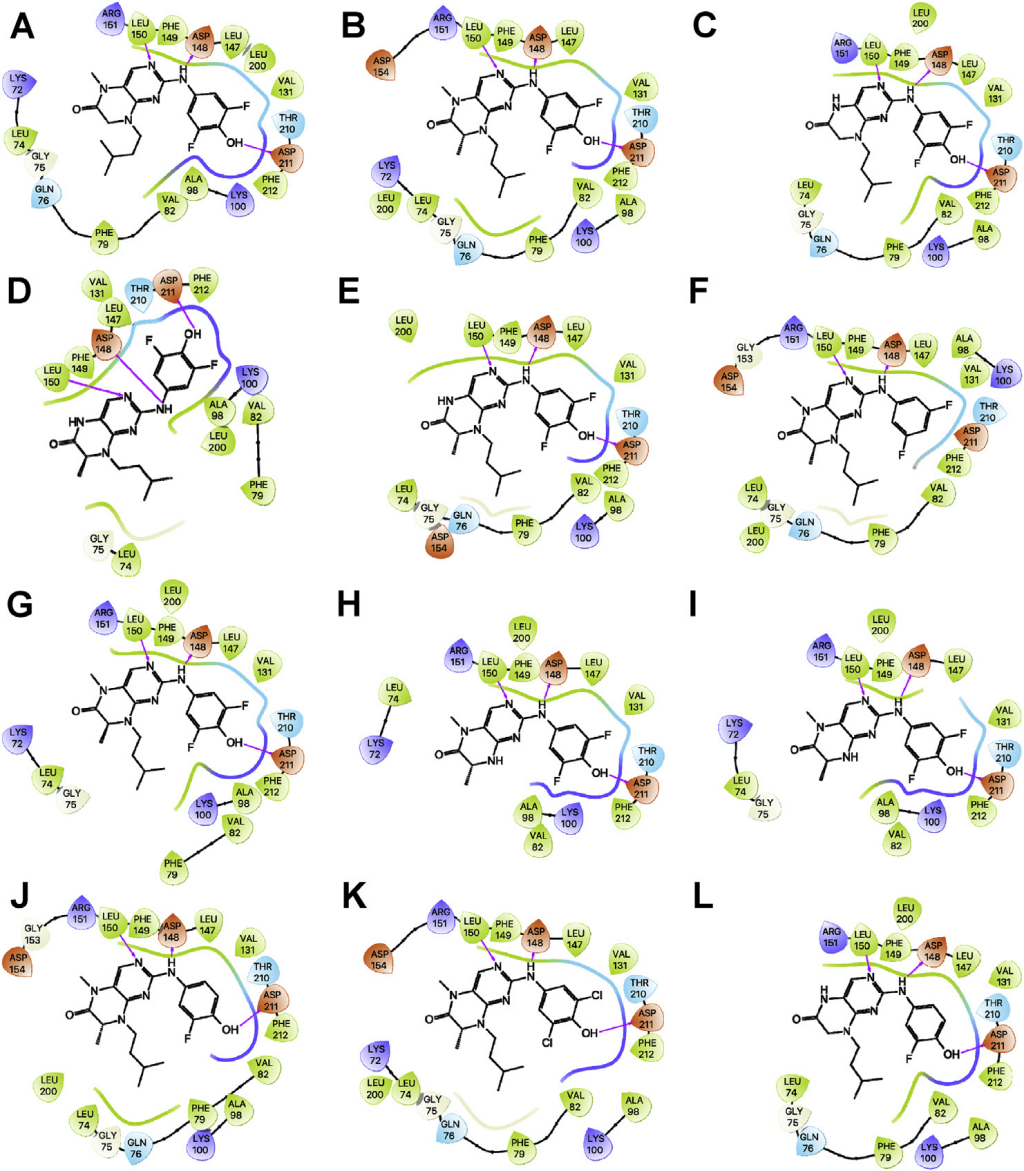
Ligand interaction map of the predicted binding mode of A) **34**, B) BI-D1870 *R*-isomer, C) **36**, D) **33***S*-isomer, E) **33***R*-isomer, F) **24***R*-isomer, G) BI-D1870 *S*-isomer, H) **39***R*-isomer, I) **39***S*-isomer, J) **28***R*-isomer, K) **31***R*-isomer, and L) **35** in the ATP-binding site of the RSK2 NTKD, where red residues are charged negative, purple residues are charged positive, green residues are hydrophobic, and blue residues are polar, magenta arrows indicate H-bonds, violet lines indicate slat bridges, and gray spheres represent areas of solvent exposure.

**Fig. 4:**
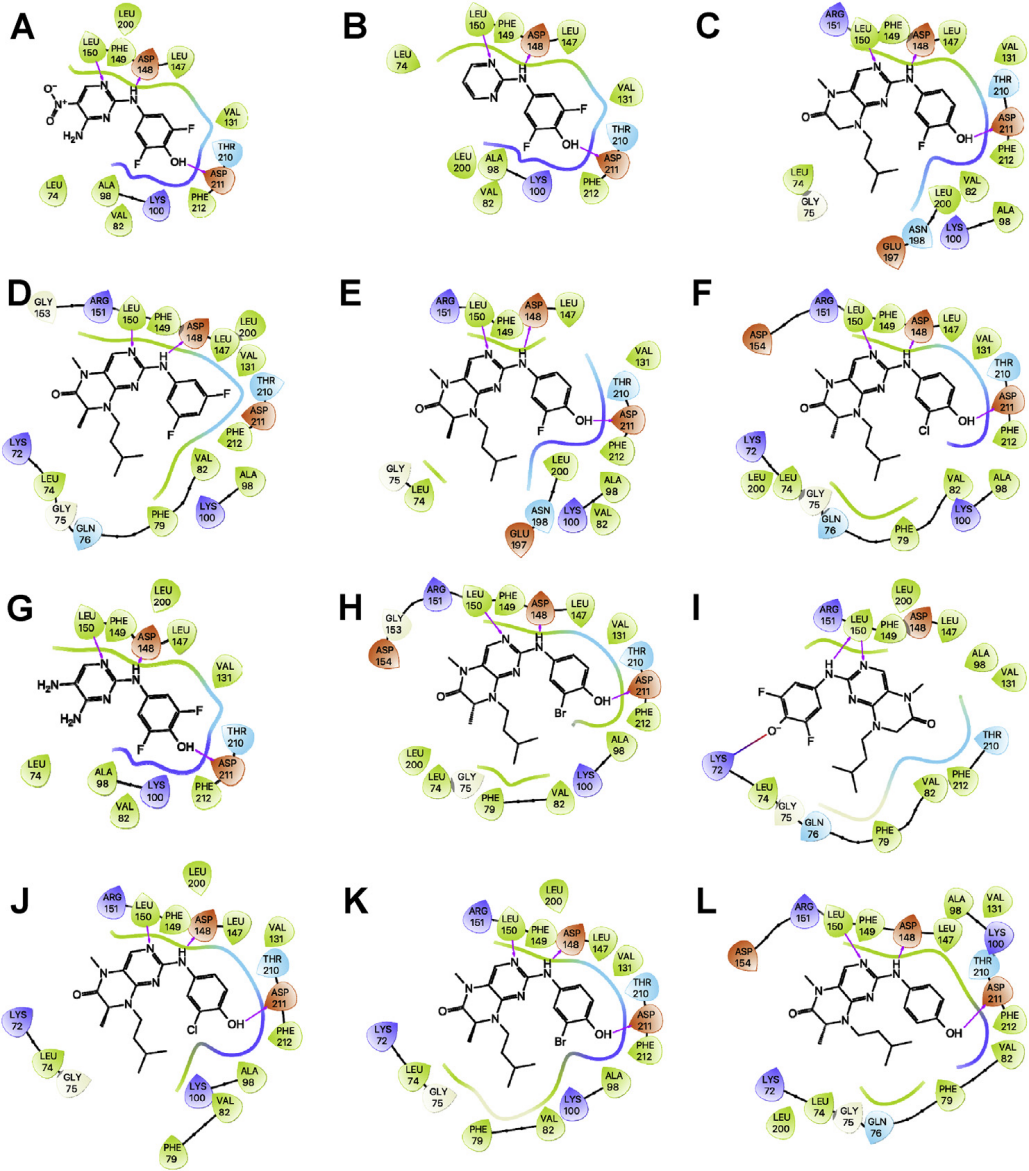
Ligand interaction map of the predicted binding mode of A) **43**, B) **38**, C) **37**, D) **24***S*-isomer, E) **28***S*-isomer, F) **29***R*-isomer, G) **44**, H) **30***R*-isomer, I) **34** deprotonated, J) **29***S*-isomer, K) **30***S*-isomer, and L) **27***R*-isomer in the ATP-binding site of the RSK2 NTKD, where red residues are charged negative, purple residues are charged positive, green residues are hydrophobic, and blue residues are polar, magenta arrows indicate H-bonds, violet lines indicate slat bridges, and gray spheres represent areas of solvent exposure.

**Fig. 5:**
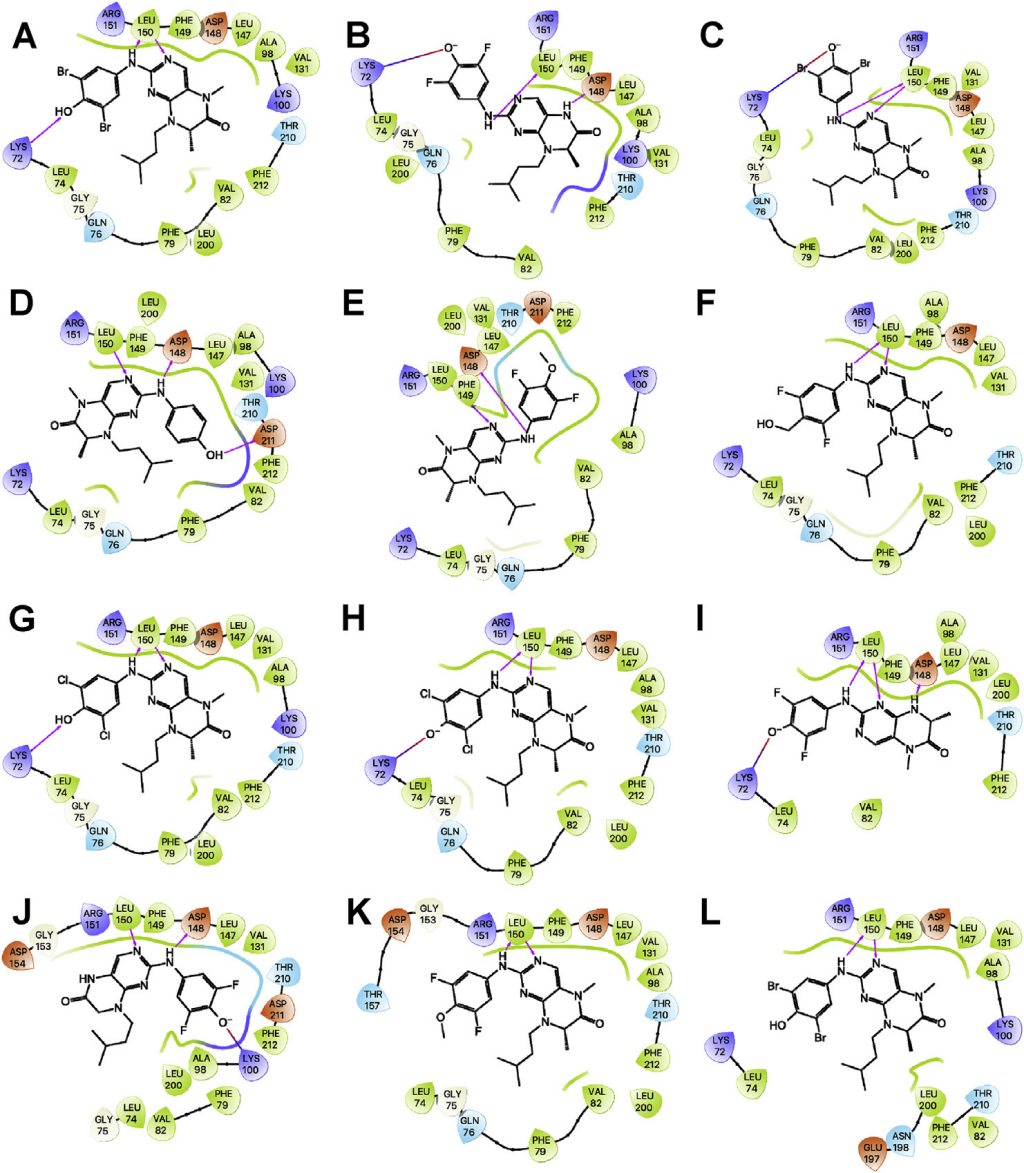
Ligand interaction map of the predicted binding mode of A) **32***S*-isomer, B) **33***R*-isomer deprotonated, C) **33***S*-isomer deprotonated, D) **27***S*-isomer, E) **25***R*-isomer, F) **26***S*-isomer, G) **31***S*-isomer, H) **31***S*-isomer deprotonated, I) **39***R*-isomer deprotonated, J) **35** deprotonated, K) **25***S*-isomer, and L) **32***R*-isomer in the ATP-binding site of the RSK2 NTKD, where red residues are charged negative, purple residues are charged positive, green residues are hydrophobic, and blue residues are polar, magenta arrows indicate H-bonds, violet lines indicate slat bridges, and gray spheres represent areas of solvent exposure.

**Fig. 6:**
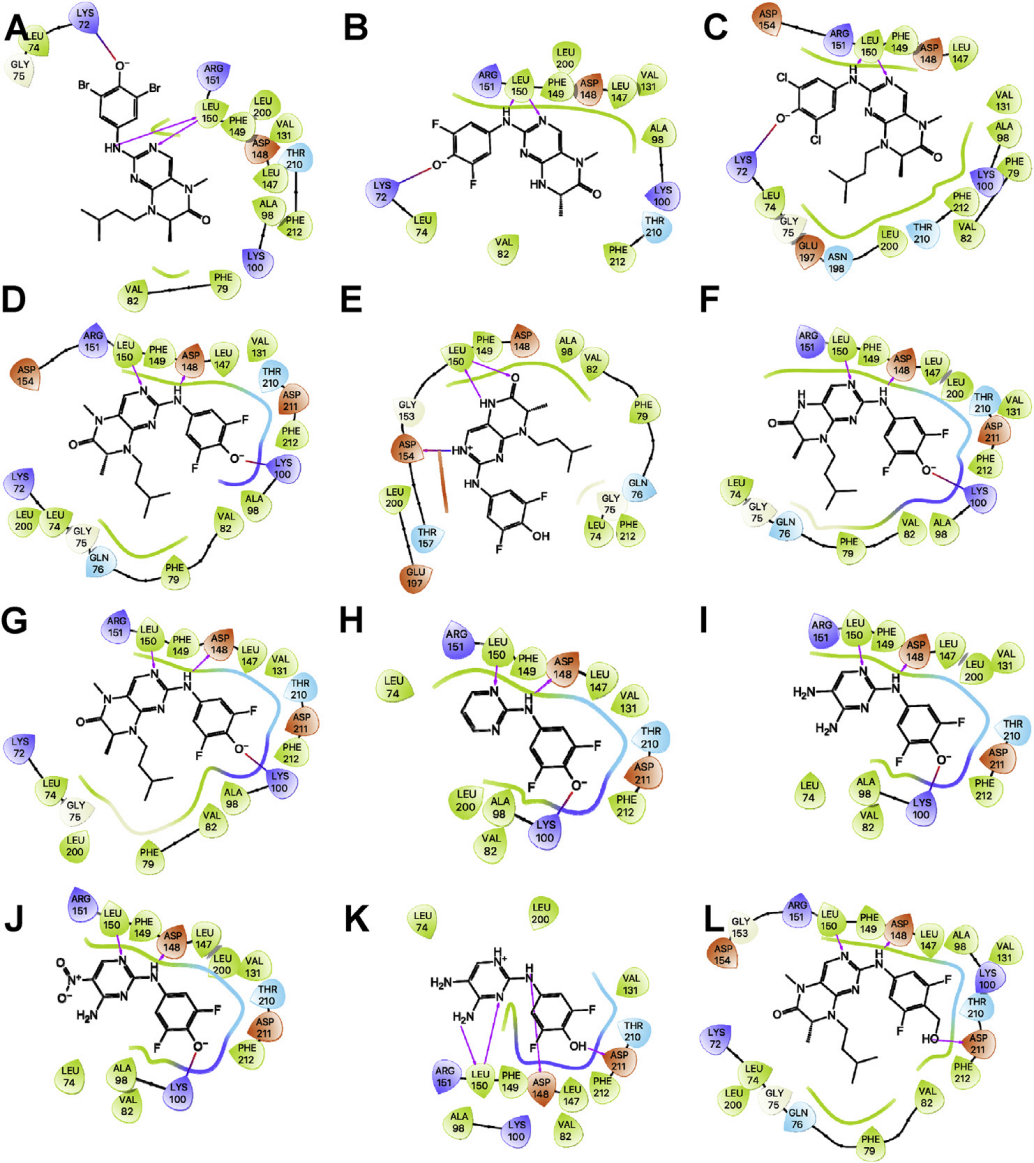
Ligand interaction map of the predicted binding mode of A) **32***R*-isomer deprotonated, B) **39***S*-isomer deprotonated, C) **31***R*-isomer deprotonated, D) BI-D1870 *R*-isomer deprotonated, E) **33***S*-isomer protonated, F) **33***S*-isomer deprotonated, G) BI-D1870 *S*-isomer deprotonated, H) **38** deprotonated, I) **44** deprotonated, J) **43** deprotonated, K) **44** protonated, and L) **26***R*-isomer in the ATP-binding site of the RSK2 NTKD, where red residues are charged negative, purple residues are charged positive, green residues are hydrophobic, and blue residues are polar, magenta arrows indicate H-bonds, violet lines indicate slat bridges, and gray spheres represent areas of solvent exposure.

**Fig. 7:**
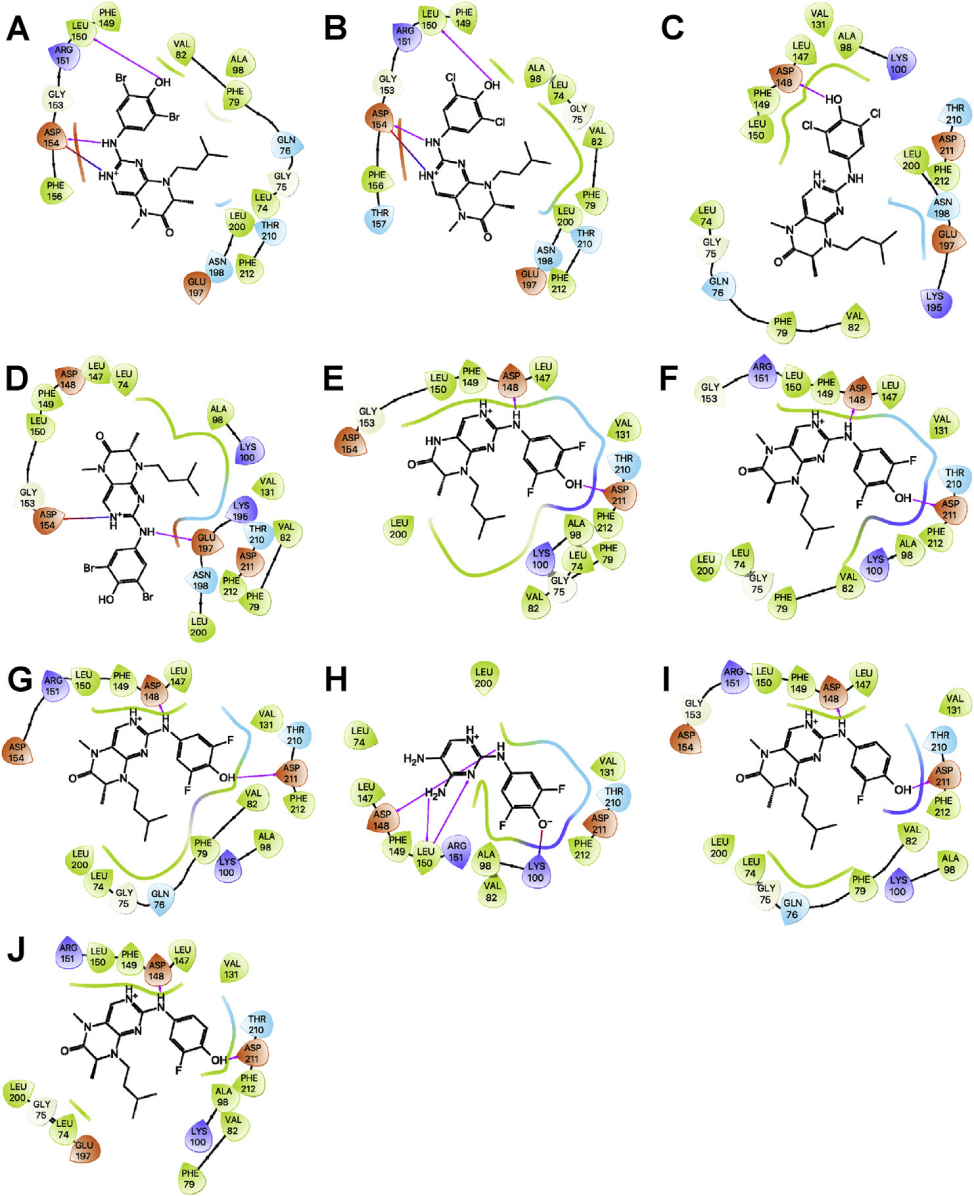
Ligand interaction map of the predicted binding mode of A) **32***R*-isomer protonated, B) **31***R*-isomer protonated, C) **31***S*-isomer deprotonated, D) **32***S*-isomer protonated, E) **33***R*-isomer protonated, F) BI-D1870 *S*-isomer protonated, G) BI-D1870 *R*-isomer protonated, H) **44** protonated/deprotonated, I) **28***R*-isomer protonated, and J) **28***S*-isomer protonated in the ATP-binding site of the RSK2 NTKD, where red residues are charged negative, purple residues are charged positive, green residues are hydrophobic, and blue residues are polar, magenta arrows indicate H-bonds, violet lines indicate slat bridges, and gray spheres represent areas of solvent exposure.

**Fig. 8:**
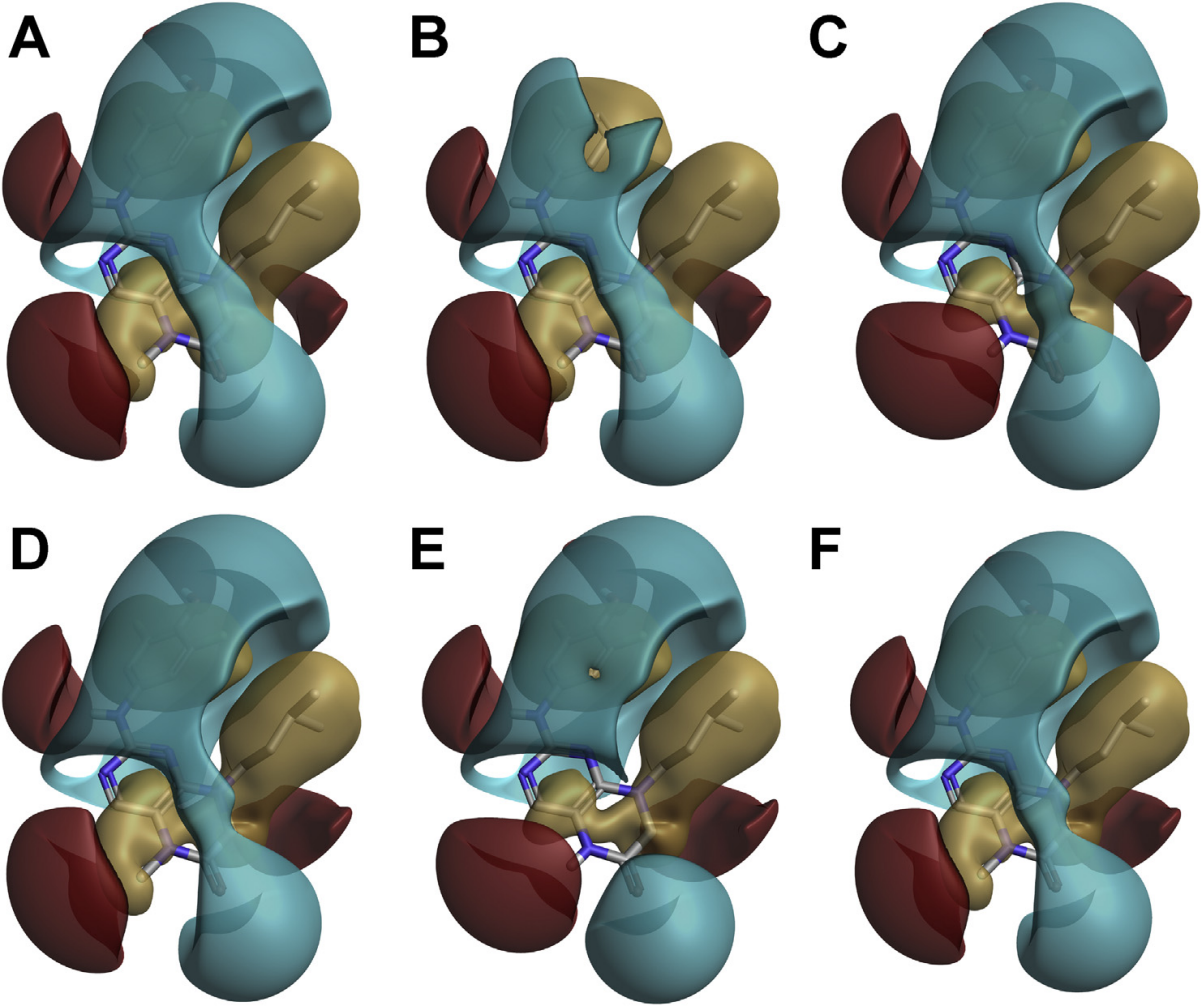
The docked conformations of a selection of compounds aligned with BI-D1870 with the calculated molecular field characteristics for each compound A) BI-D1870, B) **24**, C) **33**, D) **34**, E) **36**, and F) **37** from Cresset Forge. Hydrophobic fields are tan, negative electrostatic fields are blue, and positive electrostatic fields are red.

The 90 kDa ribosomal S6 kinase family of proteins (RSK1-4) is a group of highly conserved Ser/Thr kinases that regulate diverse cellular processes [1]. The activity of RSK2 has emerged as an attractive target for cancer therapy due to its role in the regulation of diverse cellular processes, such as cell transformation and proliferation and the maintenance of cancer stem cells (CSCs) [1]. Several pan-RSK inhibitors have been identified that target either the catalytic N-terminal kinase domain (NTKD) or activating C-terminal kinase domain (CTKD) of the RSKs [1]. Due to their high sequence homology there are no isoform-selective RSK inhibitors. The pteridinone, BI-D1870 is an ATP-competitive, potent, and frequently used small molecule pan-RSK inhibitor targeting the NTKD, that has been used to identify the physiological substrates and functional roles for RSK in cells [2]. The translational development of BI-D1870 as an anticancer agent has been impeded by its poor pharmacokinetic profile [3,4]. In order support a medicinal chemistry campaign to develop novel RSK inhibitors with improved pharmacokinetic properties, we designed and synthesized a series of pteridinones and pyrimidines (Fig. 1), to evaluate the structural features of BI-D1870 that are required for RSK2 inhibition. Here, we provide the computational-based docking parameters and outputs for all the pteridinones and pyrimidines evaluated in our study (Fig. 2, Fig. 3, Fig. 4, Fig. 5, Fig. 6, Fig. 7) and their associated calculated MM/GBSA outputs (Table 1). Furthermore, we also provide the results of a molecular field analysis of the compounds (Fig. 8). Our studies provide important protein-ligand interaction information for the further development of RSK inhibitors.

**Table 1:** The ranking of compounds from Glide docking and their calculated MM/GBSA binding energies (kcal/mol) using Prime of substituted pyrimidines and pteridinones in the ATP-binding site of the NTKD of RSK2. Inhibitory activity of compounds in the TR-FRET kinase assay against RSK2 (methods described in Casalvieri et al., 2020) given as the half-maximal inhibitory concentrations (IC_50_) or percentage inhibition; values are the mean ± S.D. (n = 3).

Compound	Rank from Glide output	Dock Score	Calculated MM/GBSA binding energy (kcal/mol)	RSK2 Inhibition
BI-D1870 *R*-isomer	2	−10.6	−86.3	23.3 ± 8.2 nM
BI-D1870 *S*-isomer	7	−10.1	−79.9	
BI-D1870 *R*-isomer deprotonated	40	−7.0	−61.8	
BI-D1870 *S*-isomer deprotonated	43	−6.8	−57.5	
BI-D1870 *R*-isomer protonated	55	−1.3	−31.5	
BI-D1870 *S*-isomer protonated	54	−1.3	−27.3	
**24***R*-isomer	6	−10.1	−80.8	71% @ 10 μM
**24***S*-isomer	16	−9.7	−83.0	
**25***R*-isomer	29	−8.0	−63.4	43% @ 10 μM
**25***S*-isomer	35	−7.3	−67.1	
**26***R*-isomer	48	−5.7	−69.0	739 ± 14.1 nM
**26***S*-isomer	30	−8.0	−67.9	
**27***R*-isomer	24	−8.6	−80.8	54.8 ± 1.4 nM
**27***S*-isomer	28	−8.1	−76.5	
**28***R*-isomer	10	−10.0	−82.4	25.4 ± 3.2 nM
**28***S*-isomer	17	−9.4	−74.6	
**28***R*-isomer protonated	57	−0.6	−31.7	
**28***S*-isomer protonated	58	−0.6	−25.2	
**29***R*-isomer	18	−9.3	−85.1	71.5 ± 10.3 nM
**29***S*-isomer	22	−8.7	−78.1	
**30***R*-isomer	20	−8.8	−80.7	141 ± 25.7 nM
**30***S*-isomer	23	−8.6	−80.9	
**31***R*-isomer	11	−9.9	−90.7	83% @ 10 μM
**31***S*-isomer	31	−7.9	−78.1	
**31***R*-isomer deprotonated	39	−7.0	−69.4	
**31***S*-isomer deprotonated	32	−7.9	−66.1	
**31***R*-isomer protonated	50	−2.8	−56.5	
**31***S*-isomer protonated	51	−2.8	−45.1	
**32***R*-isomer	36	−7.2	−69.0	78% @ 10 μM
**32***S*-isomer	25	−8.4	−78.2	
**32***R*-isomer deprotonated	37	−7.1	−74.5	
**32***S*-isomer deprotonated	27	−8.1	−71.8	
**32***R*-isomer protonated	49	−3.1	−60.6	
**32***S*-isomer protonated	52	−2.7	−47.5	
**33***R*-isomer	5	−10.3	−80.3	18.2 ± 1.4 nM
**33***S*-isomer	4	−10.3	−81.1	
**33***R*-isomer deprotonated	26	−8.4	−60.5	
**33***S*-isomer deprotonated	42	−6.9	−53.6	
**33***R*-isomer protonated	53	−2.0	−25.1	
**33***S*-isomer protonated	41	−7.0	−48.1	
**34**	1	−10.8	−81.3	17.6 ± 1.4 nM
**34** deprotonated	21	−8.8	−69.9	
**35**	12	−9.9	−77.1	38.3 ± 7.9 nM
**35** deprotonated	34	−7.3	−50.5	
**36**	3	−10.5	−81.8	23.4 ± 4.7 nM
**37**	15	−9.8	−76.9	24.7 ± 1.8 nM
**38**	14	−9.8	−54.0	45% @ 10 μM
**38** deprotonated	44	−6.6	−28.9	
**39***R*-isomer	8	−10.0	−66.5	83% @ 10 μM
**39***S*-isomer	9	−10.0	−66.7	
**39***R*-isomer deprotonated	33	−7.6	−58.5	
**39***S*-isomer deprotonated	38	−7.1	−58.7	
**43**	13	−9.8	−58.9	72% @ 10 μM
**43** deprotonated	46	−6.2	−34.5	
**44**	19	−9.0	−53.2	38% @ 10 μM
**44** deprotonated	45	−6.2	−29.4	
**44** protonated	47	−5.9	−21.7	
**44** deprotonated/protonated	56	−0.7	−0.1

## 2 Experimental design, materials, and methods

### 2.1 Molecular docking and binding energy determination

Schrödinger (Release 2018-4, Schrödinger LLC, New York, NY, https://www.schrodinger.com) Glide was used to dock the series of pyrimidines and pteridinones to the ATP-binding site of the NTKD of RSK2 and Prime was used to calculate the associated MM/GBSA energies.

#### 2.1.1 Glide docking

All the compounds shown in Fig. 1 were prepared using LigPrep to desalt and generate all possible tautomers and states at pH 7.0 using Epik, specified chiralities were retained, and compounds were minimized using the OPLS_2005 force field [5]. The crystal structure of the NTKD of RSK2 co-crystallized with BI-D1870 (PDB: 5D9K) was obtained from the Protein Data Bank (PDB) [6]. The protein was prepared to assign bond orders, add hydrogens for pH 7.0 using Epik, remove water molecules, and Prime was used to complete missing side chains and loops, and termini were capped. To complete protein preparation a restrained minimization of the protein structure was performed using the default constraint of 0.30 Å RMSD and the OPLS_2005 force field [5]. The binding site was defined as a 6 Å region around the co-crystalized BI-D1870 defined as a single binding site region in SiteMap [7], and the receptor grid was defined based on this entry using Receptor Grid Generation. Molecular docking simulations were performed using the Glide ligand docking module in XP (extra precision) mode and included a post-docking minimization [8]. The binding conformations were examined to identify critical interactions (Fig. 3, Fig. 4, Fig. 5, Fig. 6, Fig. 7).

#### 2.1.2 Binding energy calculation

Prime MM-GBSA (Molecular Mechanics/Generalized Born Model and Solvent Accessibility) was used to estimate the ligand binding energies and ligand strain energies for the series of pyrimidines and pteridinones and RSK2, which includes the OPLS_2005 force field, VSGB solvent model [9], and rotamer search algorithms. The Prime MM-GBSA simulation was carried out by using the Glide pose viewer file to calculate the total free energy of binding. The MM/GBSA calculations were performed to estimate the relative binding affinity of ligands to the receptor. The MM/GBSA calculations are used to estimate relative binding affinity of ligands to the receptor (reported in kcal/mol). As the MM/GBSA binding energies are approximate free energies of binding, a more negative value indicates stronger binding.

### 2.2 Molecular field analysis

The molecular field characteristics for each of the compounds were calculated and aligned over the bound conformation of BI-D1870 taken from the crystal structure of BI-D1870 in complex with RSK2 using Forge (10.6.0 Cresset Biomolecular Discovery Ltd, Cambridgeshire, UK, https://www.cresset-group.com/software/forge/) [10]. The *R*-isomer of each compound was used in the calculations to match the bound *R*-isomer of BI-D1870 in the crystal structure (PDB ID: 5D9K) [6]. The protein was also included in the alignment calculations as an excluded volume and the calculation method was set to Very Accurate and Slow using the default settings. A key characteristic of the most active compounds (BI-D1870, **33**, **34**, **36**, and **37**) is the extensive negative electrostatic field localized over the difluorophenol moiety (Fig. 8). Substitutions that resulted in the attenuation of this field, observed with **24** (Fig. 8), dramatically reduced inhibitory potency against RSK2. In contrast, enhancement of the positive electrostatic field over the pteridinone core, such as in **33**, was associated with increased potency.
