# Supplementary material for: Molecular docking of substituted pteridinones and pyrimidines to the ATP-binding site of the N-terminal domain of RSK2 and associated MM/GBSA and molecular field datasets
Source: Data Brief. 2020 Feb 28;29:105347. doi: 10.1016/j.dib.2020.105347 (PMC7082523; doi:10.1016/j.dib.2020.105347)
Supplement: Multimedia component 1 [file mmc1.zip › Schrodinger/5D9K-KAC.pdf]

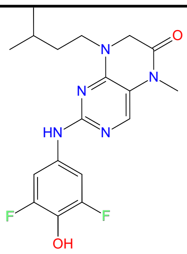 title: KAC Series 01.sdf	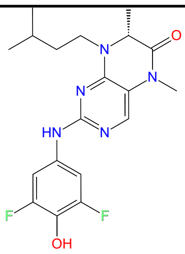 title: KAC Series 01.sdf	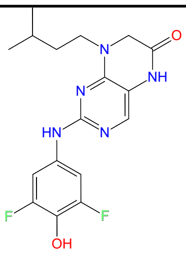 title: KAC Series 01.sdf
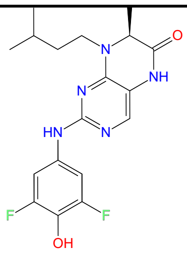 title: KAC Series 01.sdf	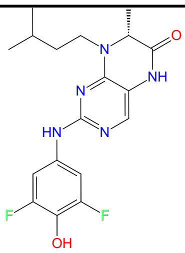 title: KAC Series 01.sdf	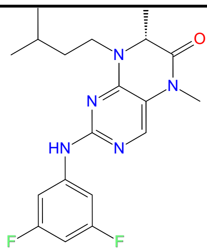 title: KAC Series 01.sdf
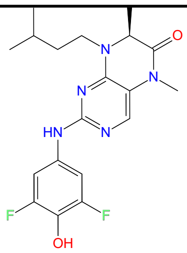 title: KAC Series 01.sdf	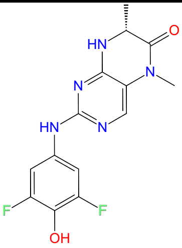 title: KAC Series 01.sdf	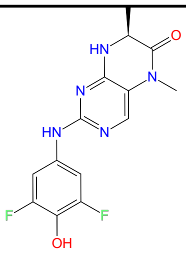 title: KAC Series 01.sdf
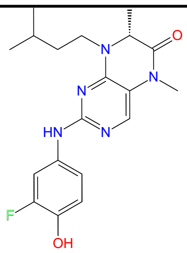 title: KAC Series 01.sdf	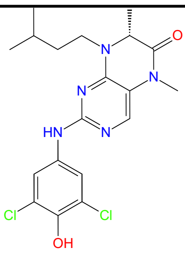 title: KAC Series 01.sdf	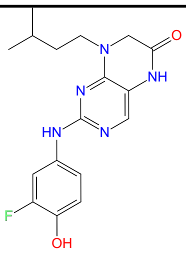 title: KAC Series 01.sdf
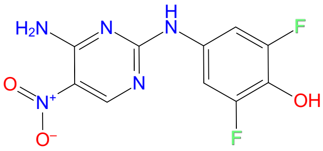 title: KAC Series 01.sdf	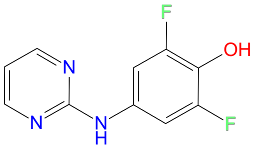 title: KAC Series 01.sdf	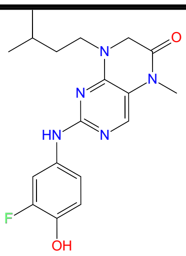 title: KAC Series 01.sdf
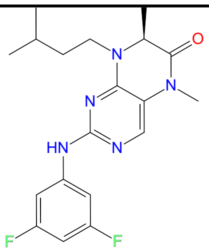 title: KAC Series 01.sdf	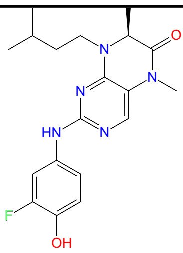 title: KAC Series 01.sdf	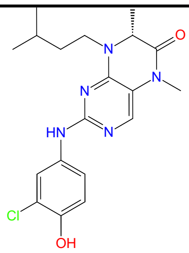 title: KAC Series 01.sdf

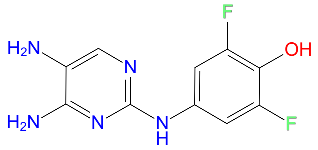

title: KAC Series 01.sdf

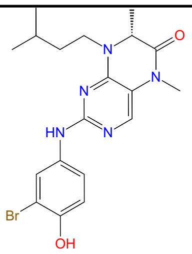

title: KAC Series 01.sdf

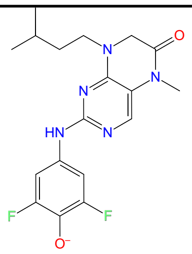

title: KAC Series 01.sdf

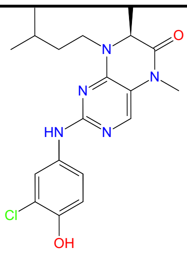

title: KAC Series 01.sdf

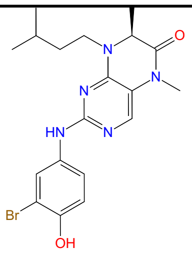

title: KAC Series 01.sdf

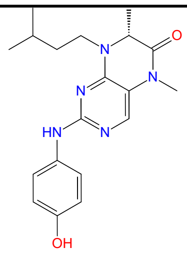

title: KAC Series 01.sdf

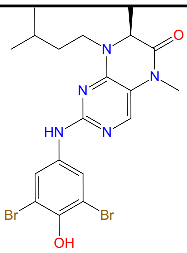

title: KAC Series 01.sdf

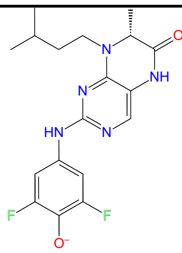

title: KAC Series 01.sdf

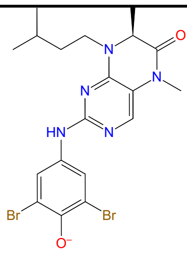

title: KAC Series 01.sdf

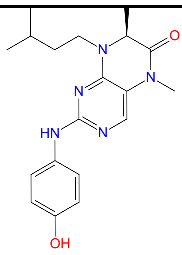

title: KAC Series 01.sdf

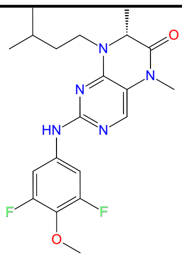

title: KAC Series 01.sdf

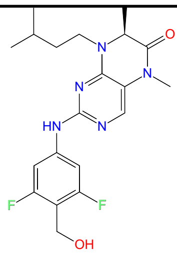

title: KAC Series 01.sdf

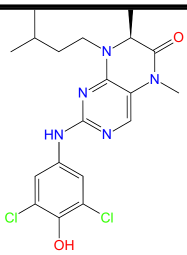

title: KAC Series 01.sdf

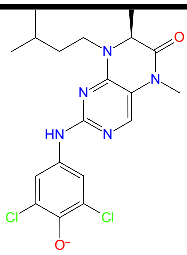

title: KAC Series 01.sdf

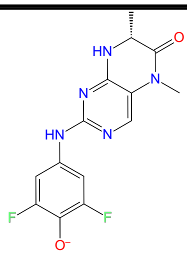

title: KAC Series 01.sdf

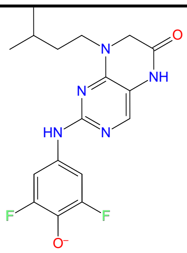

title: KAC Series 01.sdf

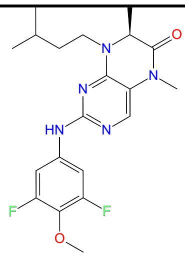

title: KAC Series 01.sdf

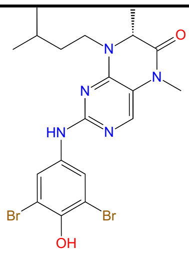

title: KAC Series 01.sdf

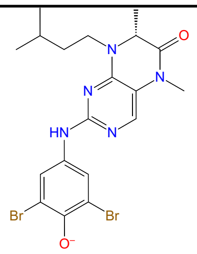

title: KAC Series 01.sdf

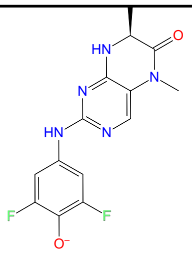

title: KAC Series 01.sdf

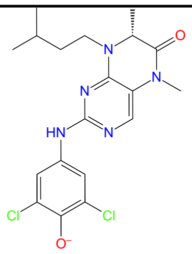

title: KAC Series 01.sdf

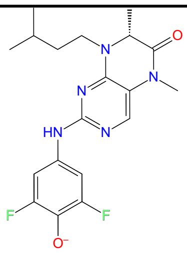

title: KAC Series 01.sdf

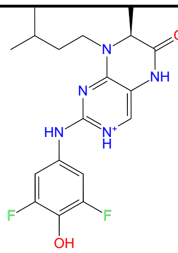

title: KAC Series 01.sdf

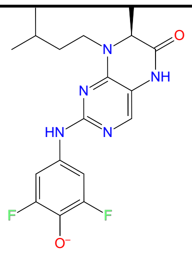

title: KAC Series 01.sdf

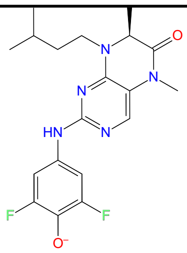

title: KAC Series 01.sdf

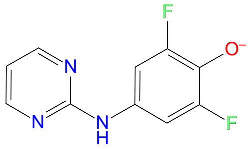

title: KAC Series 01.sdf

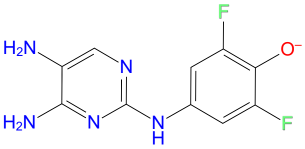

title: KAC Series 01.sdf

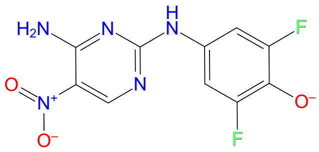

title: KAC Series 01.sdf

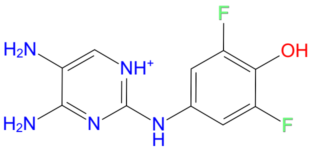

title: KAC Series 01.sdf

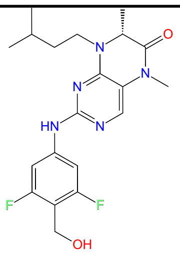

title: KAC Series 01.sdf

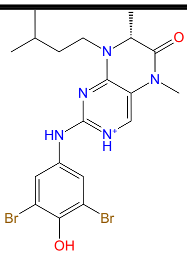

title: KAC Series 01.sdf

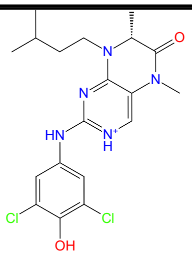

title: KAC Series 01.sdf

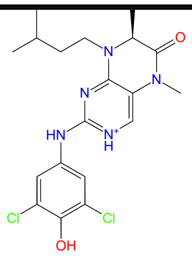

title: KAC Series 01.sdf

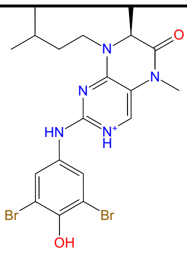

title: KAC Series 01.sdf

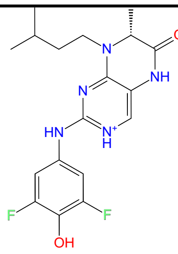

title: KAC Series 01.sdf

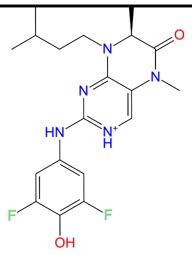

title: KAC Series 01.sdf

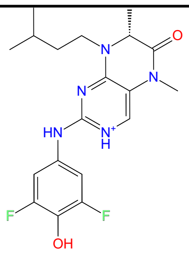

title: KAC Series 01.sdf

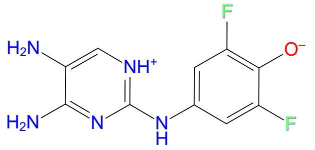

title: KAC Series 01.sdf

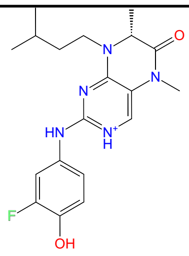

title: KAC Series 01.sdf

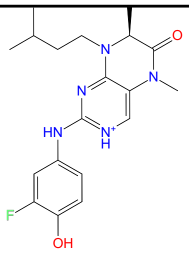

title: KAC Series 01.sdf
