# Supplementary figures and images for: Molecular docking of substituted pteridinones and pyrimidines to the ATP-binding site of the N-terminal domain of RSK2 and associated MM/GBSA and molecular field datasets
Source: Data Brief. 2020 Feb 28;29:105347. doi: 10.1016/j.dib.2020.105347 (PMC7082523; doi:10.1016/j.dib.2020.105347)

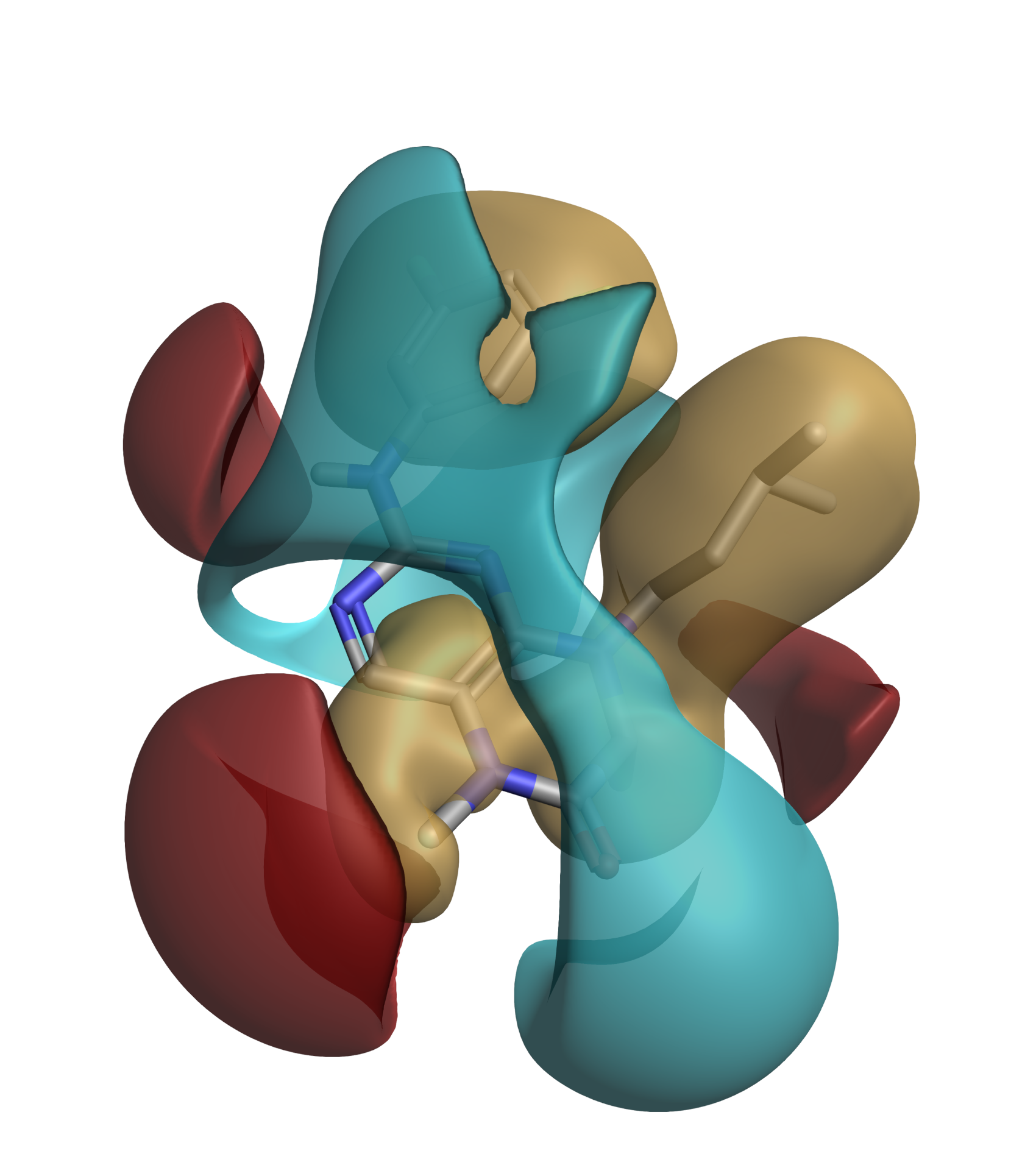

Supplement: Multimedia component 1 [file mmc1.zip › Cresset/Molecular Fields/24.png]

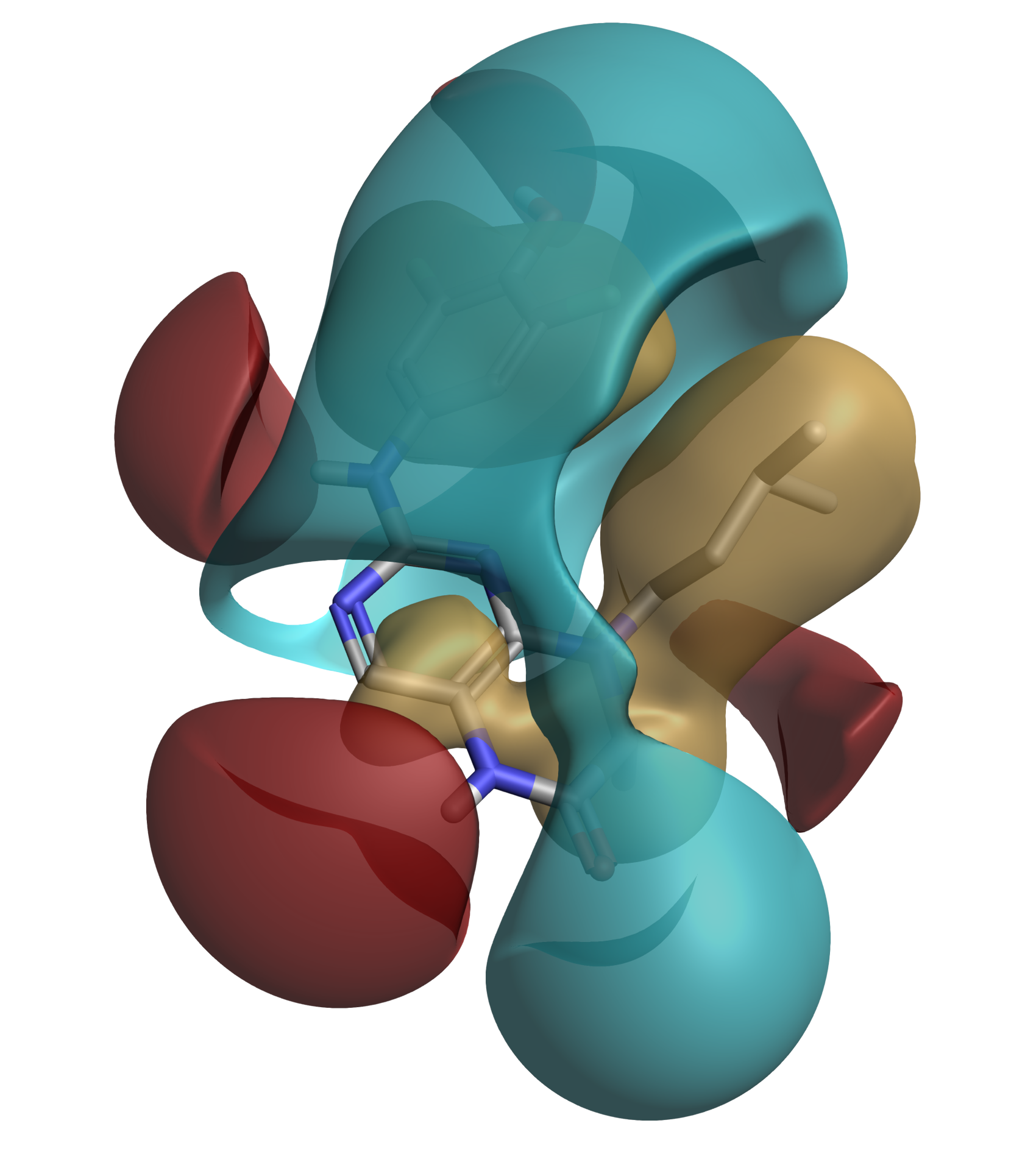

Supplement: Multimedia component 1 [file mmc1.zip › Cresset/Molecular Fields/33.png]

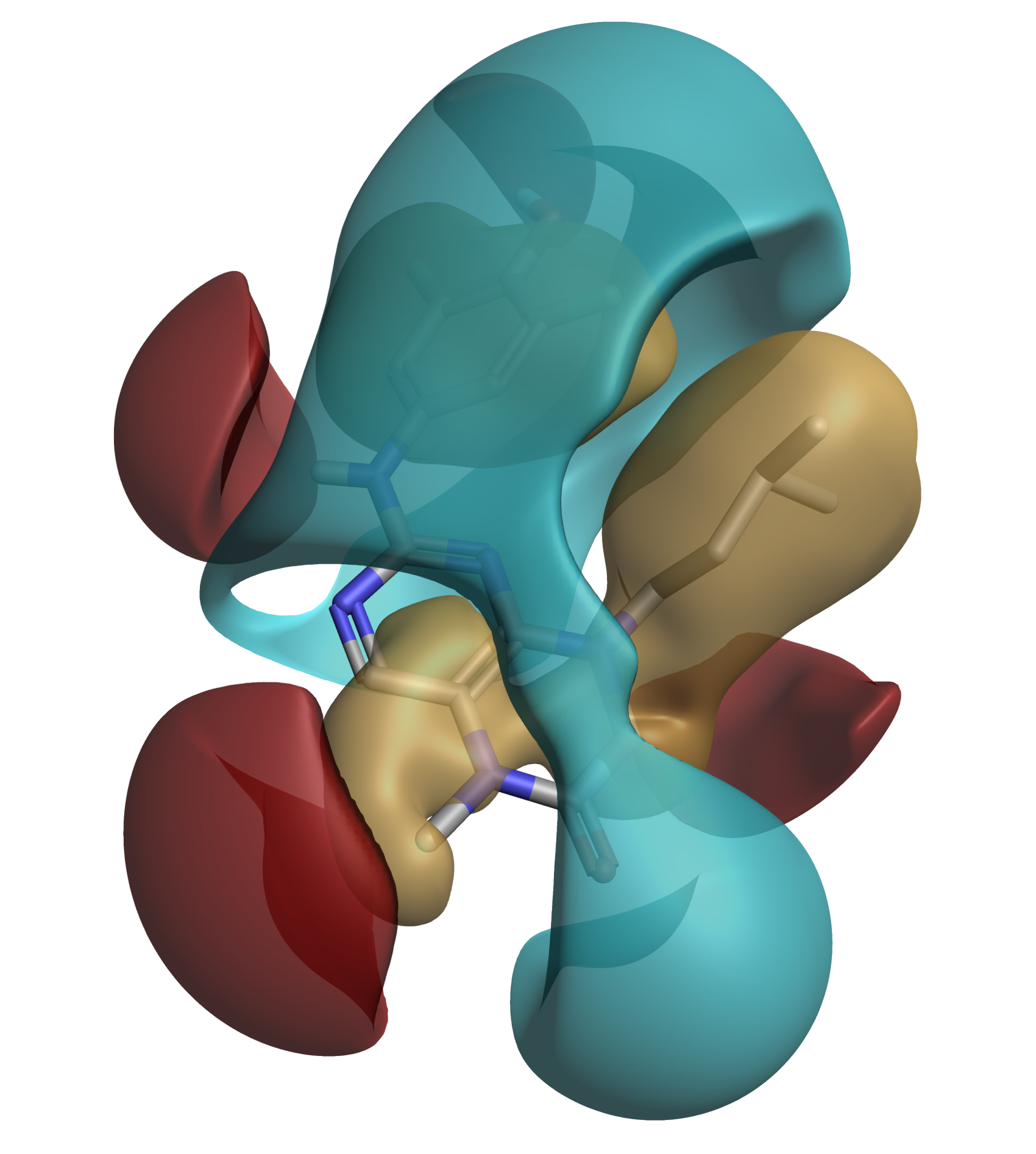

Supplement: Multimedia component 1 [file mmc1.zip › Cresset/Molecular Fields/34.png]

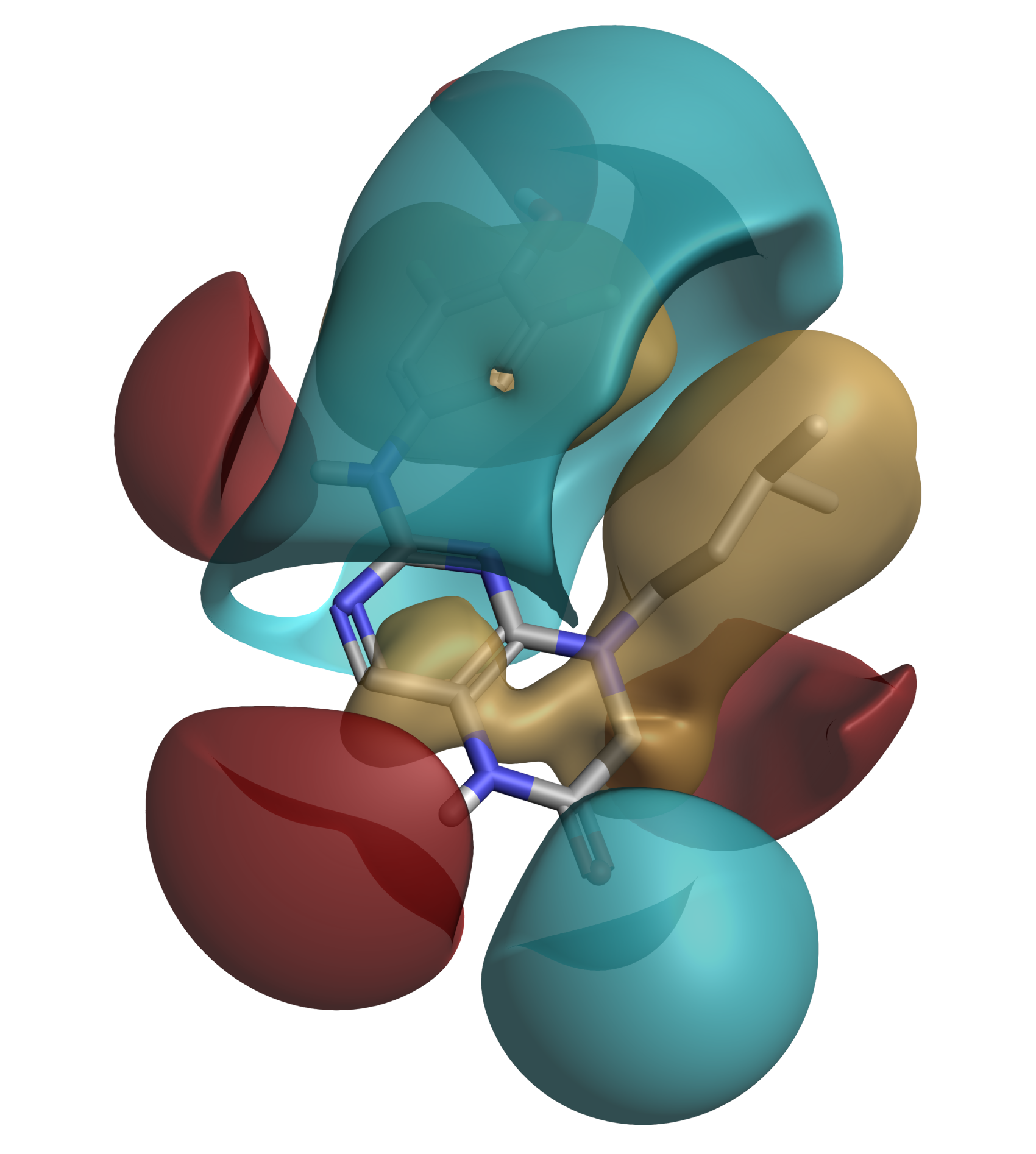

Supplement: Multimedia component 1 [file mmc1.zip › Cresset/Molecular Fields/36.png]

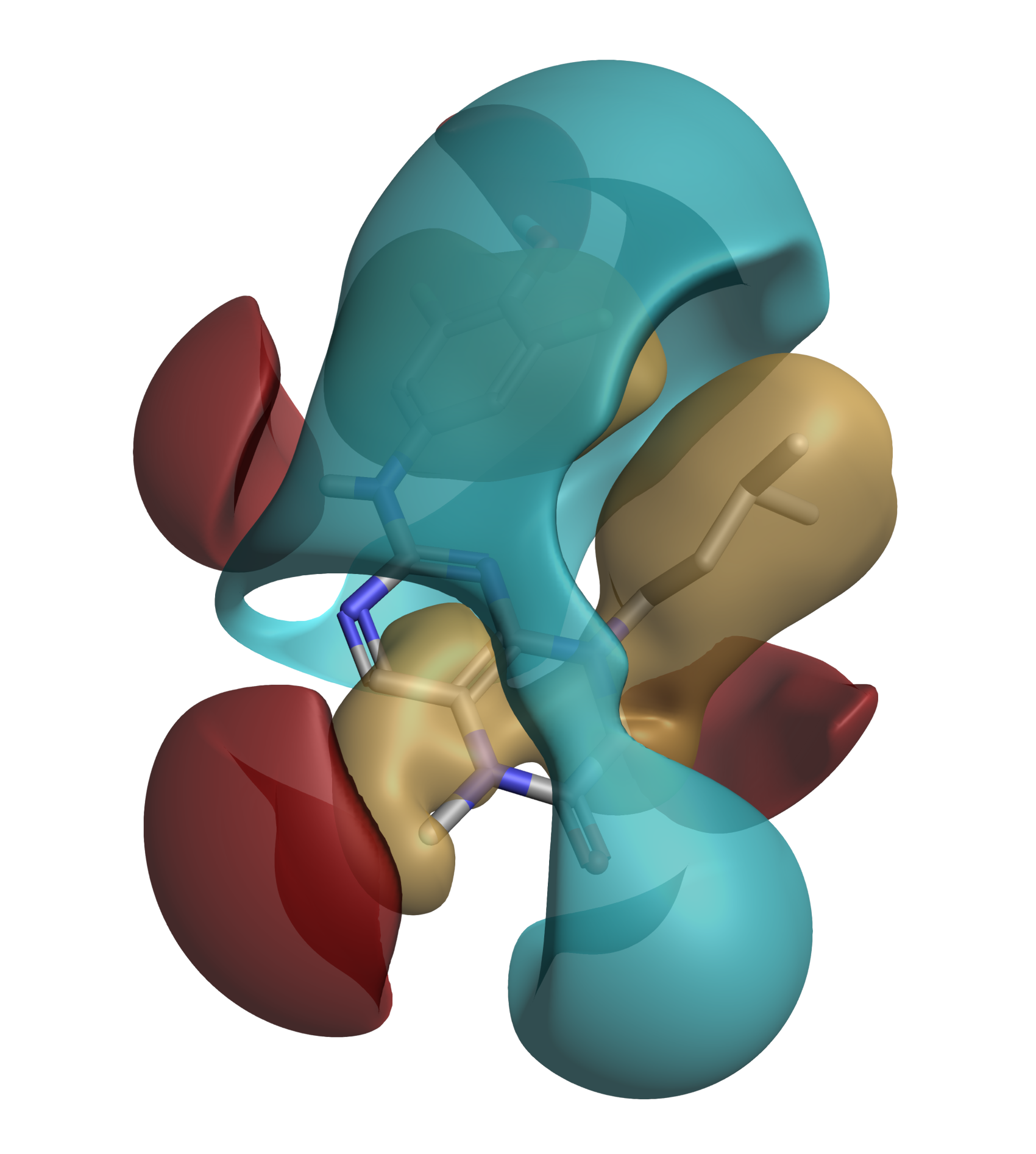

Supplement: Multimedia component 1 [file mmc1.zip › Cresset/Molecular Fields/37.png]

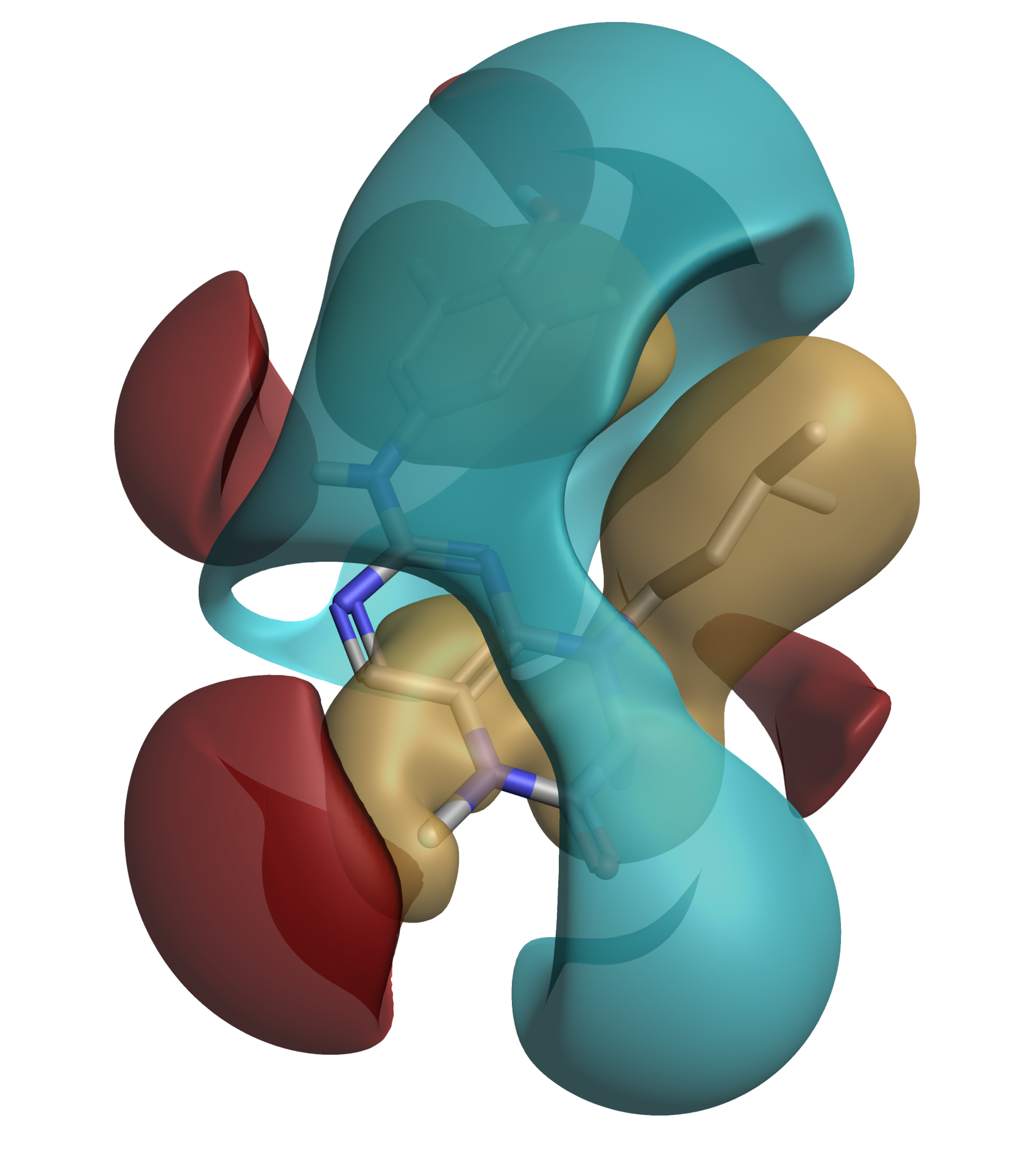

Supplement: Multimedia component 1 [file mmc1.zip › Cresset/Molecular Fields/BI-D1870.png]

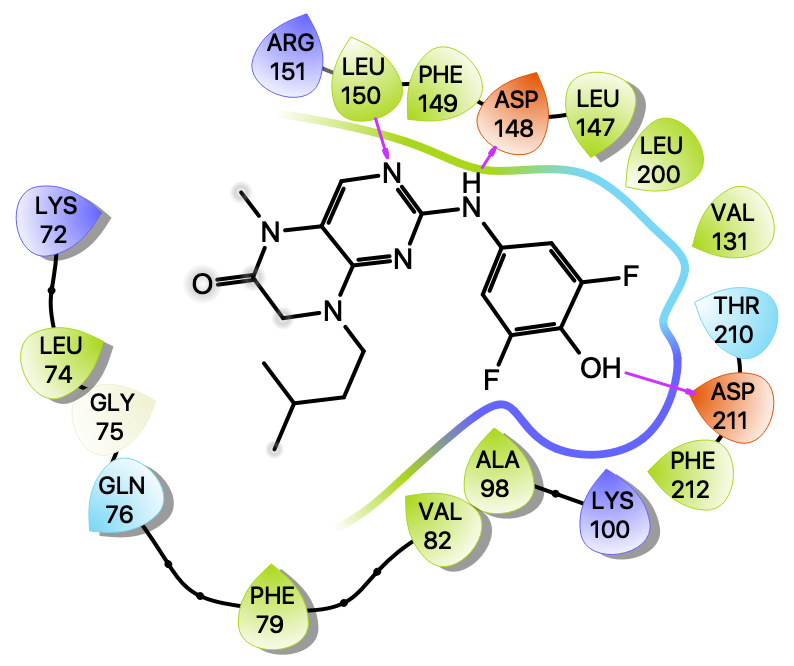

Supplement: Multimedia component 1 [file mmc1.zip › Schrodinger/Ligand Interaction Maps/01.png]

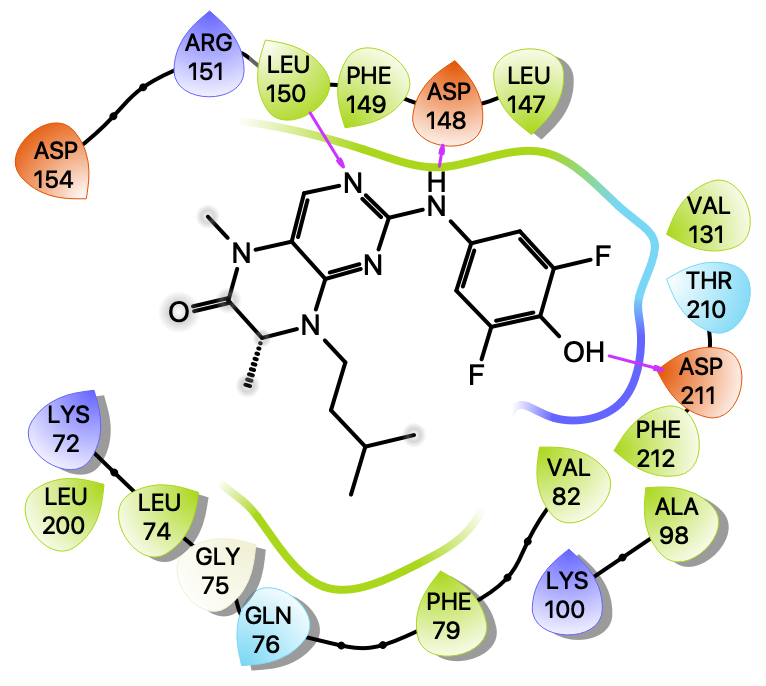

Supplement: Multimedia component 1 [file mmc1.zip › Schrodinger/Ligand Interaction Maps/02.png]

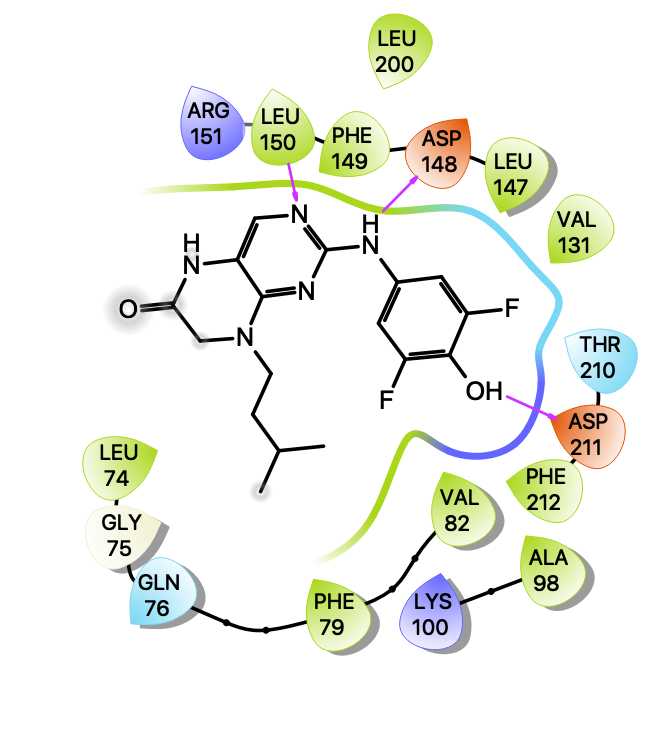

Supplement: Multimedia component 1 [file mmc1.zip › Schrodinger/Ligand Interaction Maps/03.png]

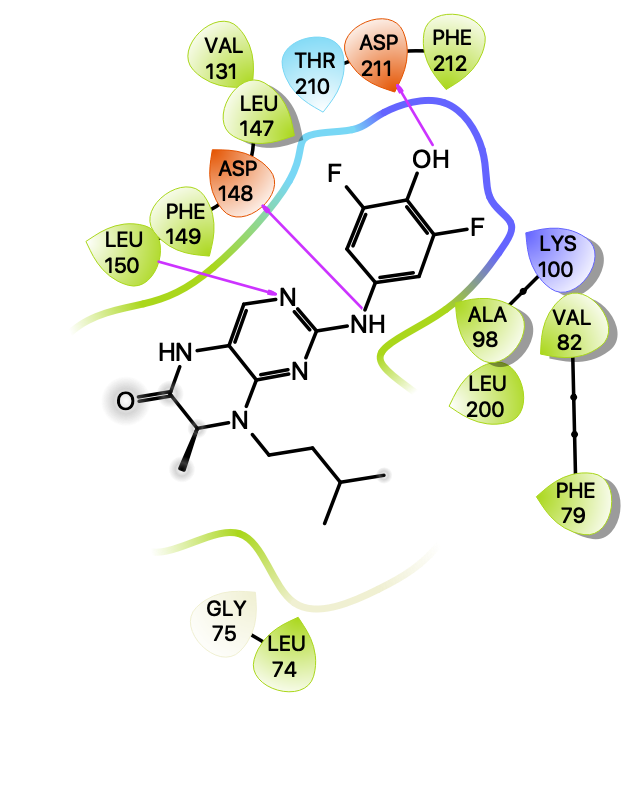

Supplement: Multimedia component 1 [file mmc1.zip › Schrodinger/Ligand Interaction Maps/04.png]

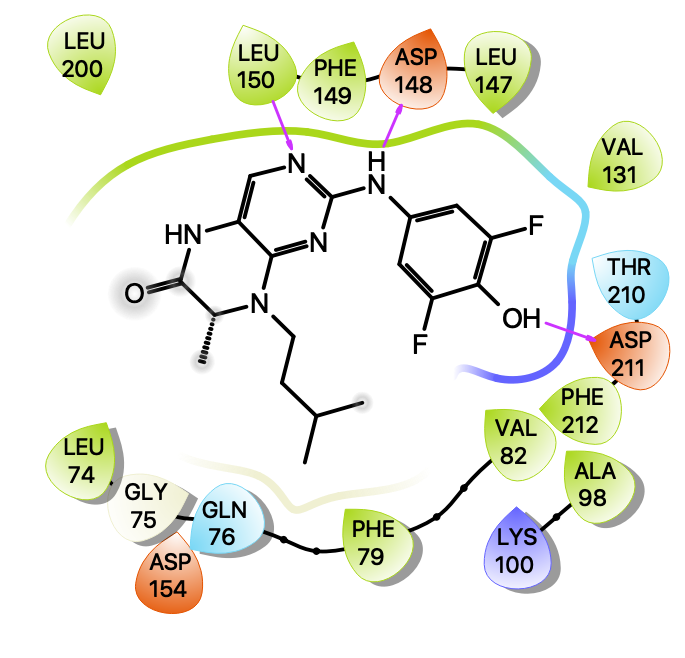

Supplement: Multimedia component 1 [file mmc1.zip › Schrodinger/Ligand Interaction Maps/05.png]

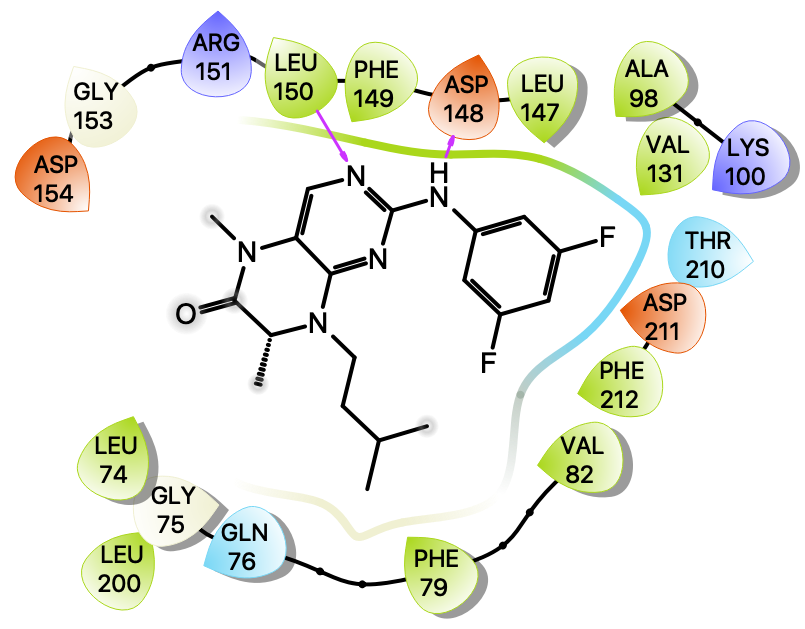

Supplement: Multimedia component 1 [file mmc1.zip › Schrodinger/Ligand Interaction Maps/06.png]

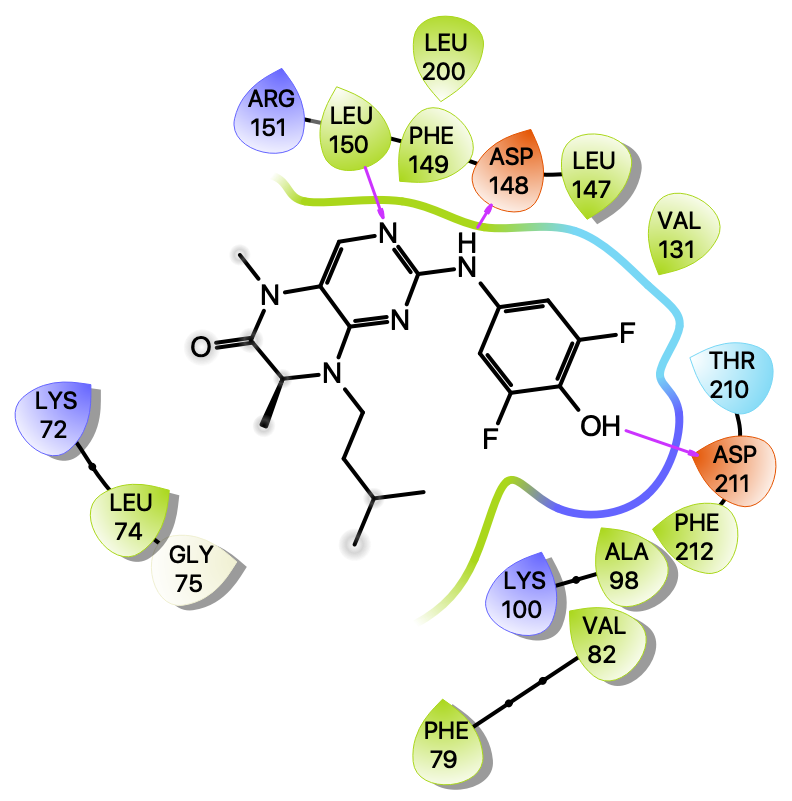

Supplement: Multimedia component 1 [file mmc1.zip › Schrodinger/Ligand Interaction Maps/07.png]

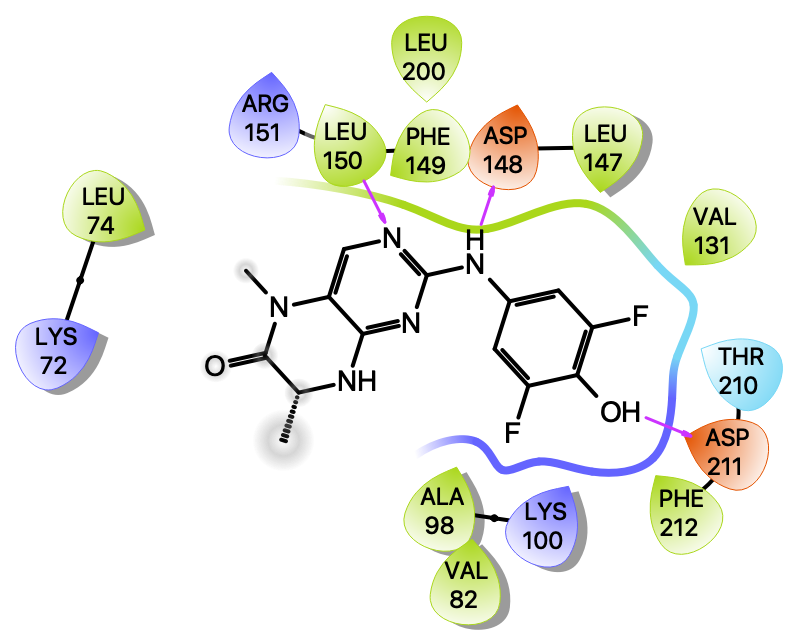

Supplement: Multimedia component 1 [file mmc1.zip › Schrodinger/Ligand Interaction Maps/08.png]

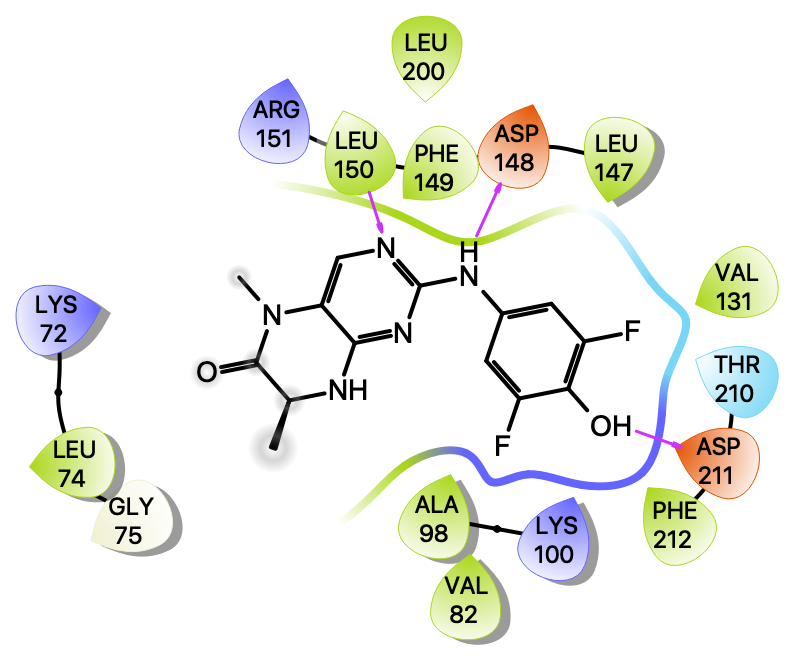

Supplement: Multimedia component 1 [file mmc1.zip › Schrodinger/Ligand Interaction Maps/09.png]

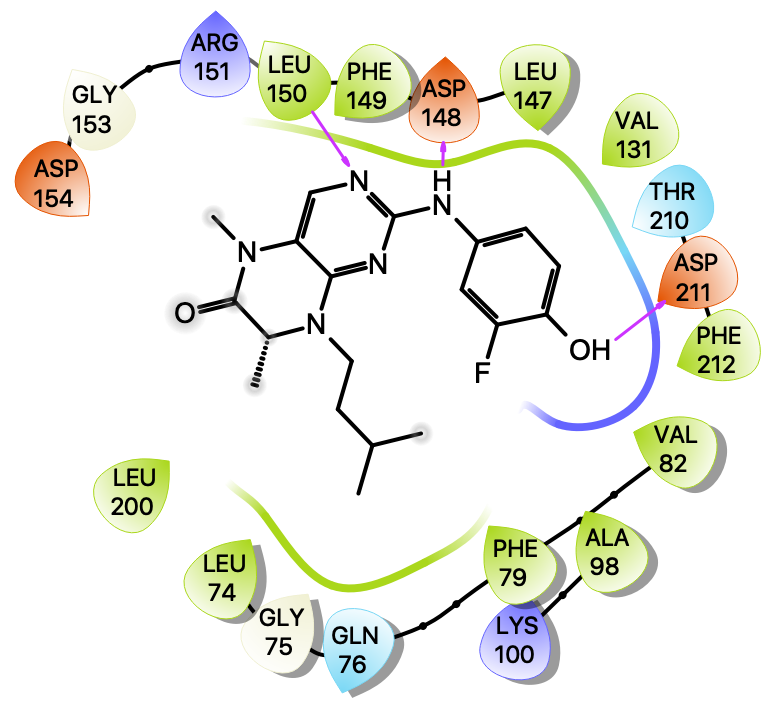

Supplement: Multimedia component 1 [file mmc1.zip › Schrodinger/Ligand Interaction Maps/10.png]

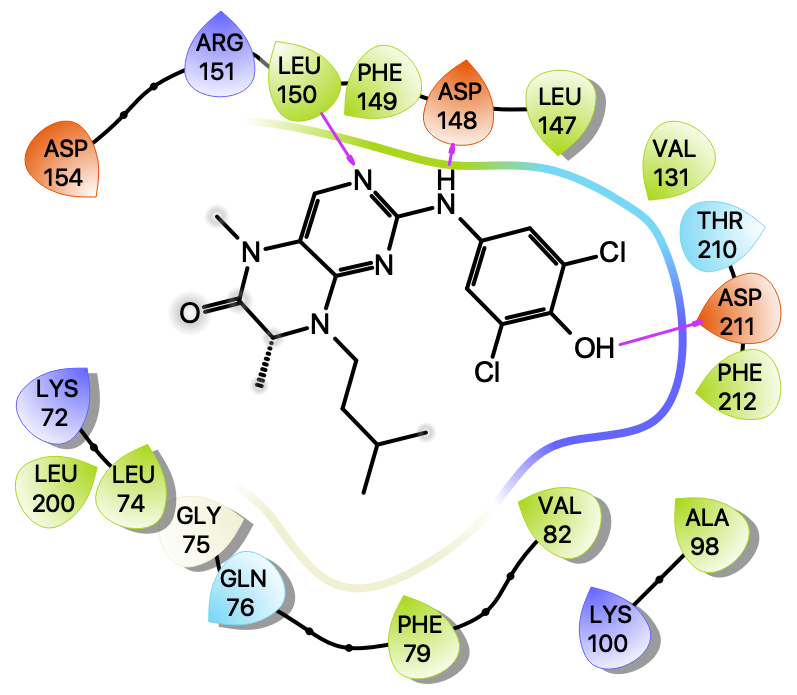

Supplement: Multimedia component 1 [file mmc1.zip › Schrodinger/Ligand Interaction Maps/11.png]

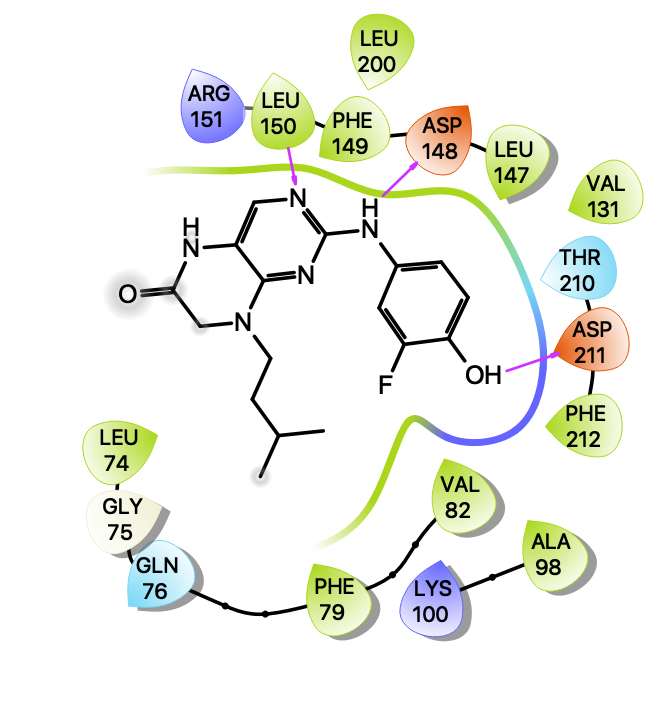

Supplement: Multimedia component 1 [file mmc1.zip › Schrodinger/Ligand Interaction Maps/12.png]

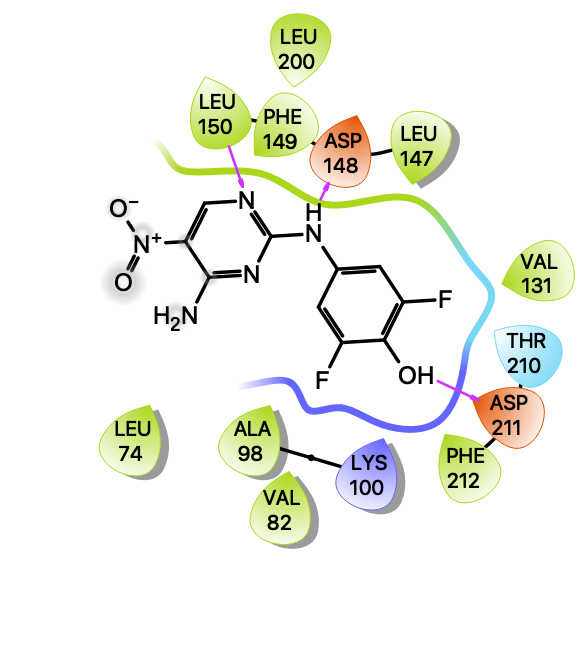

Supplement: Multimedia component 1 [file mmc1.zip › Schrodinger/Ligand Interaction Maps/13.png]

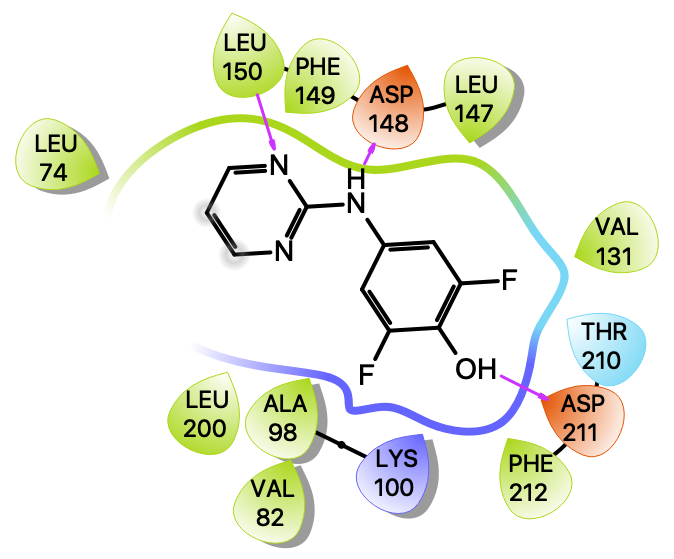

Supplement: Multimedia component 1 [file mmc1.zip › Schrodinger/Ligand Interaction Maps/14.png]

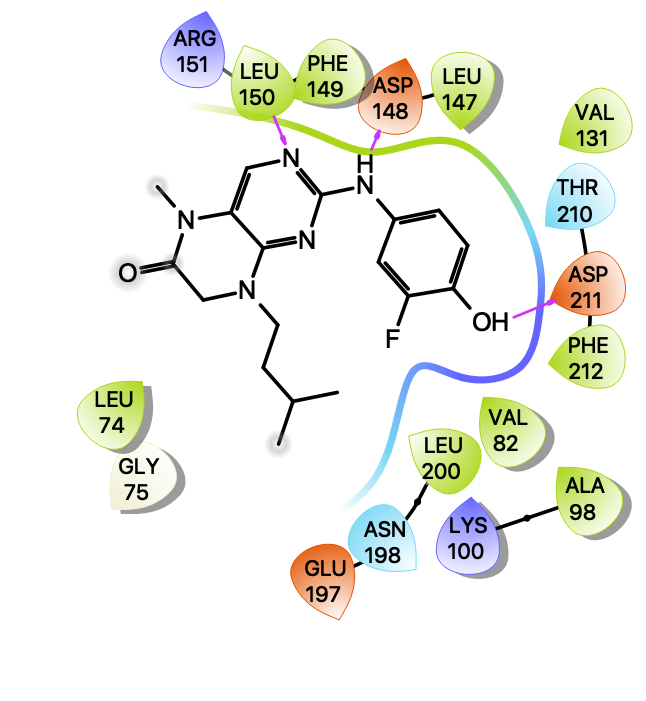

Supplement: Multimedia component 1 [file mmc1.zip › Schrodinger/Ligand Interaction Maps/15.png]

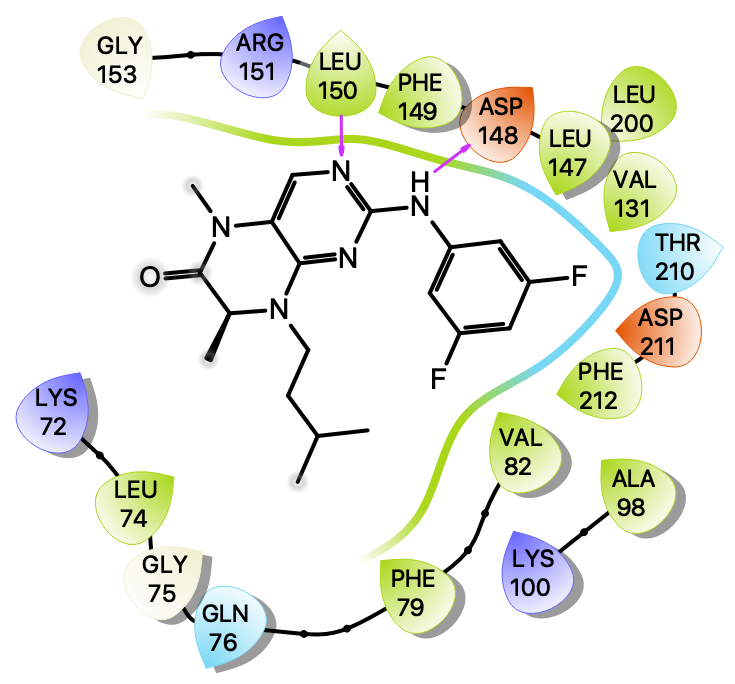

Supplement: Multimedia component 1 [file mmc1.zip › Schrodinger/Ligand Interaction Maps/16.png]

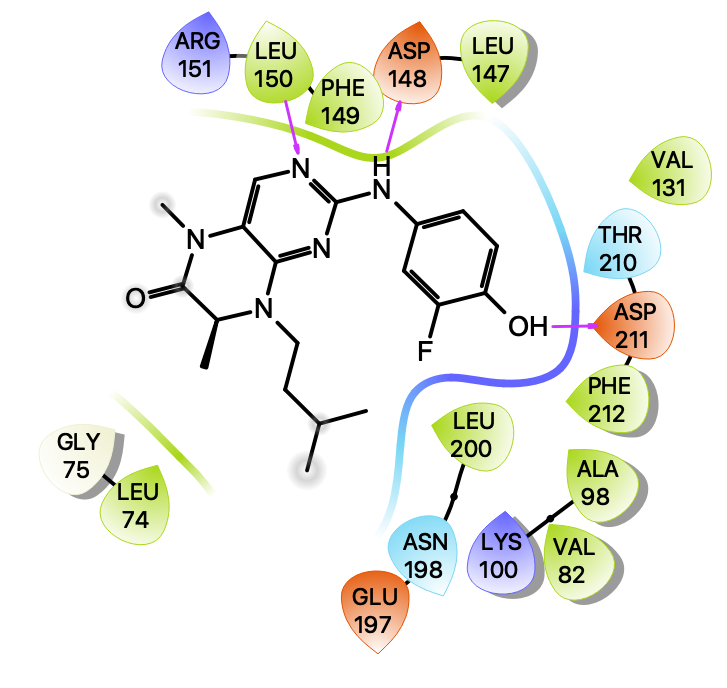

Supplement: Multimedia component 1 [file mmc1.zip › Schrodinger/Ligand Interaction Maps/17.png]

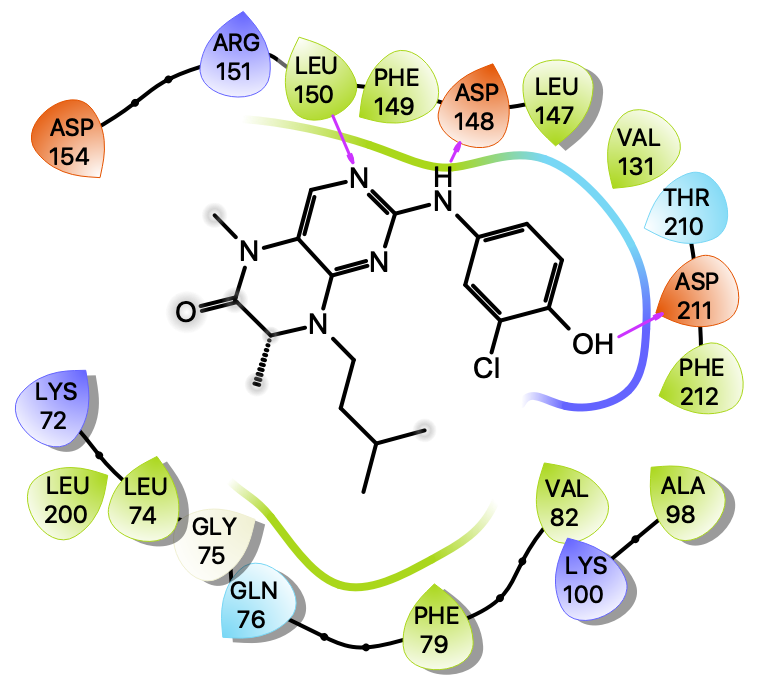

Supplement: Multimedia component 1 [file mmc1.zip › Schrodinger/Ligand Interaction Maps/18.png]

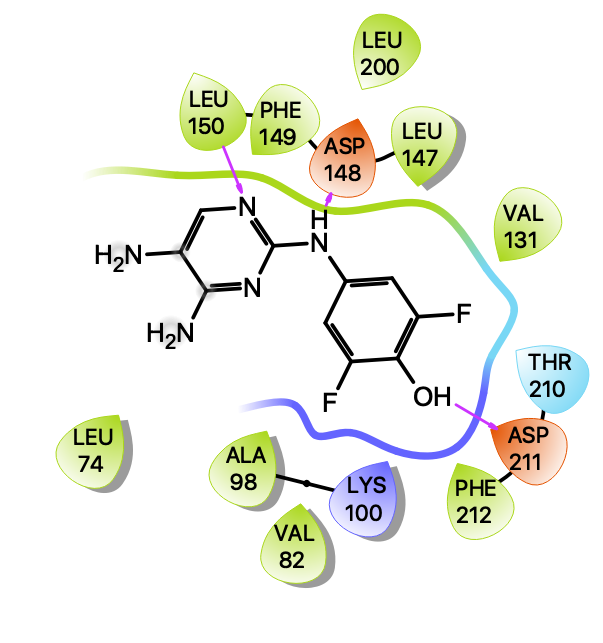

Supplement: Multimedia component 1 [file mmc1.zip › Schrodinger/Ligand Interaction Maps/19.png]

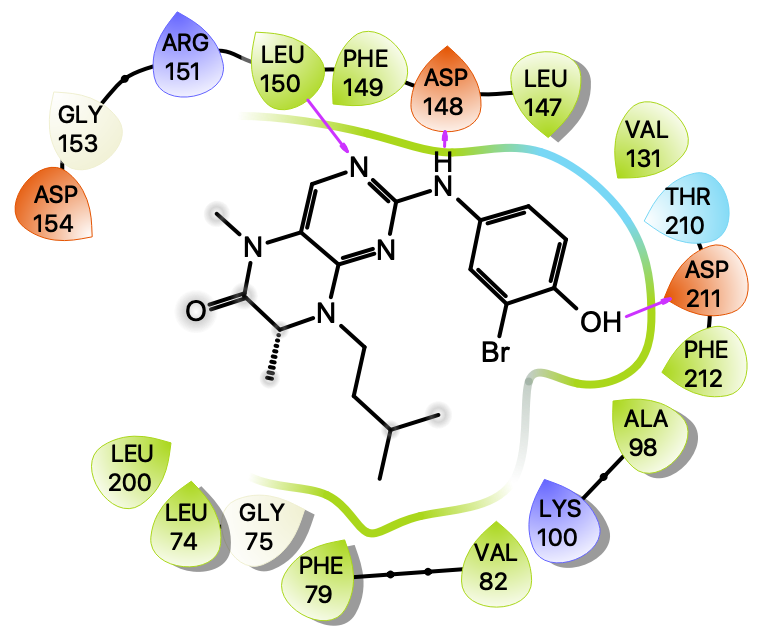

Supplement: Multimedia component 1 [file mmc1.zip › Schrodinger/Ligand Interaction Maps/20.png]

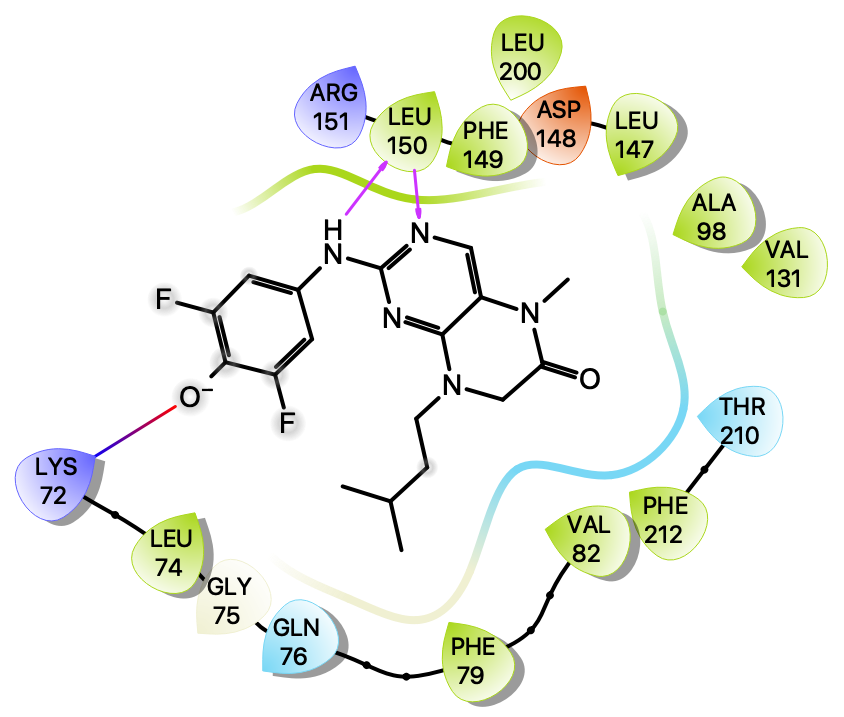

Supplement: Multimedia component 1 [file mmc1.zip › Schrodinger/Ligand Interaction Maps/21.png]

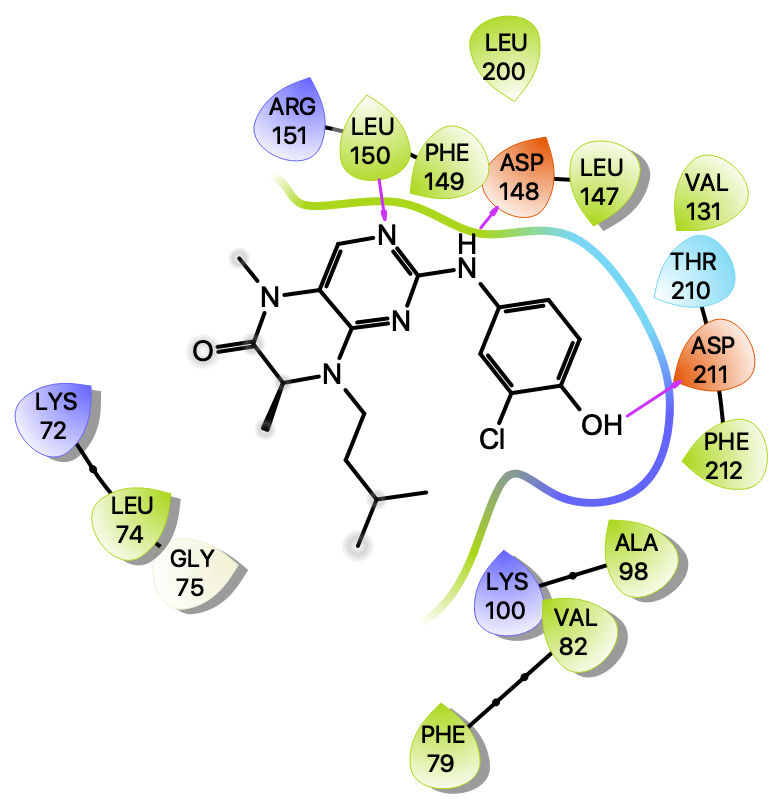

Supplement: Multimedia component 1 [file mmc1.zip › Schrodinger/Ligand Interaction Maps/22.png]

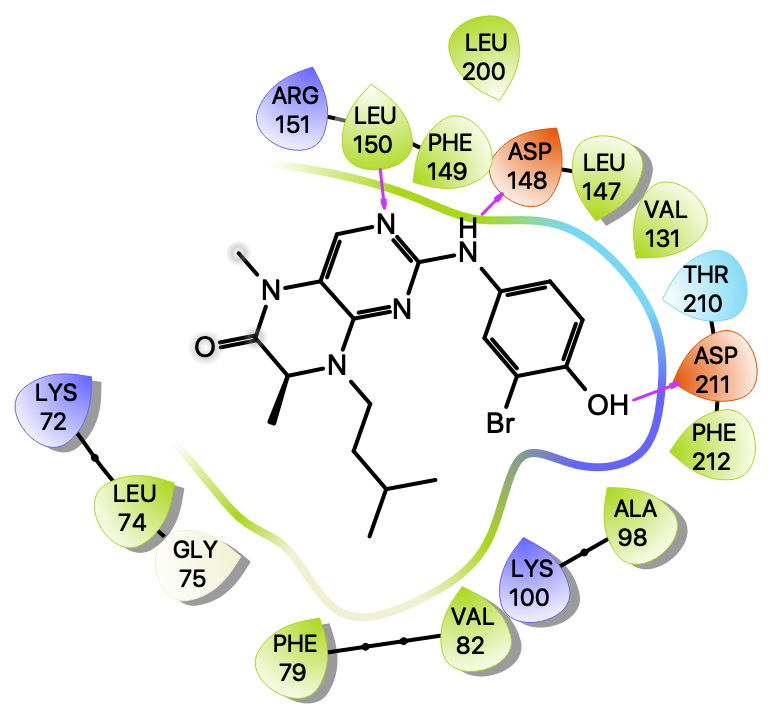

Supplement: Multimedia component 1 [file mmc1.zip › Schrodinger/Ligand Interaction Maps/23.png]

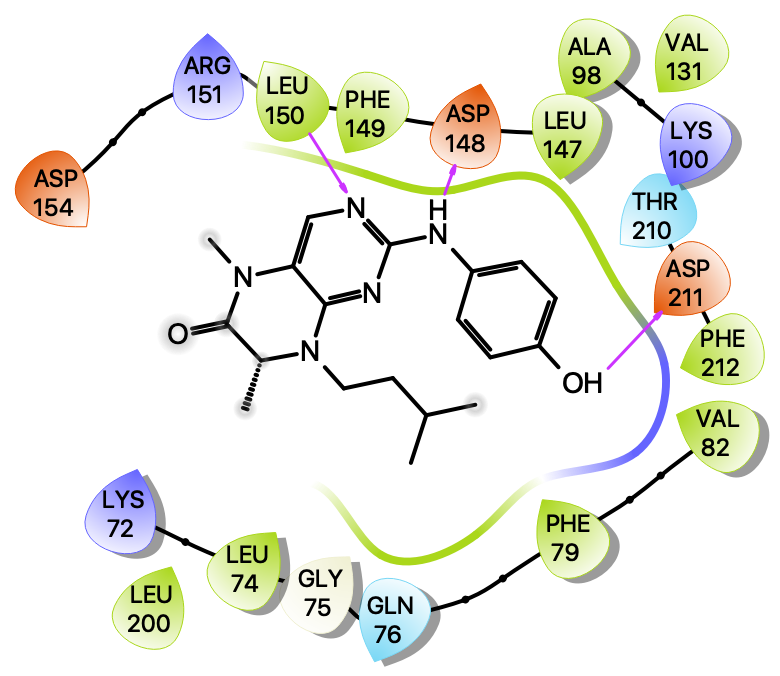

Supplement: Multimedia component 1 [file mmc1.zip › Schrodinger/Ligand Interaction Maps/24.png]

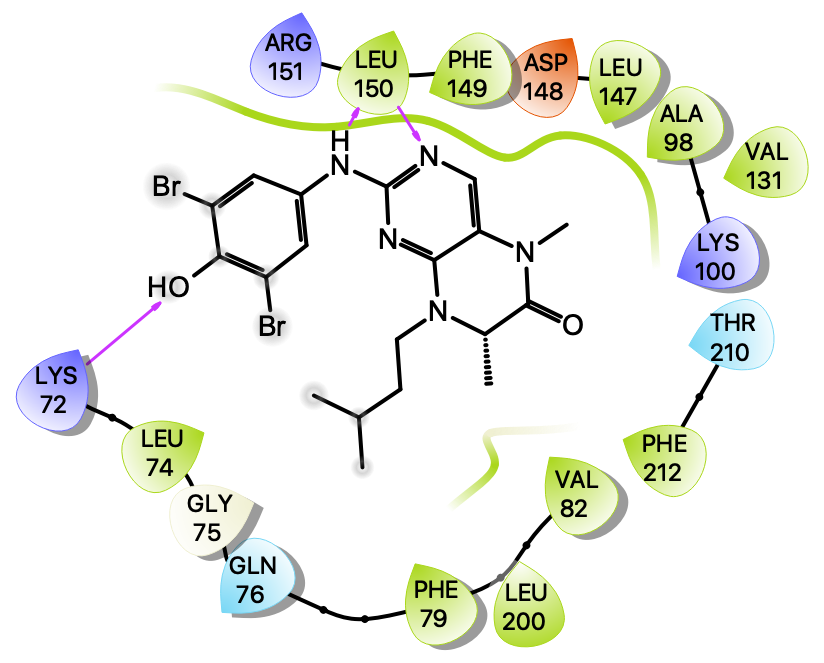

Supplement: Multimedia component 1 [file mmc1.zip › Schrodinger/Ligand Interaction Maps/25.png]

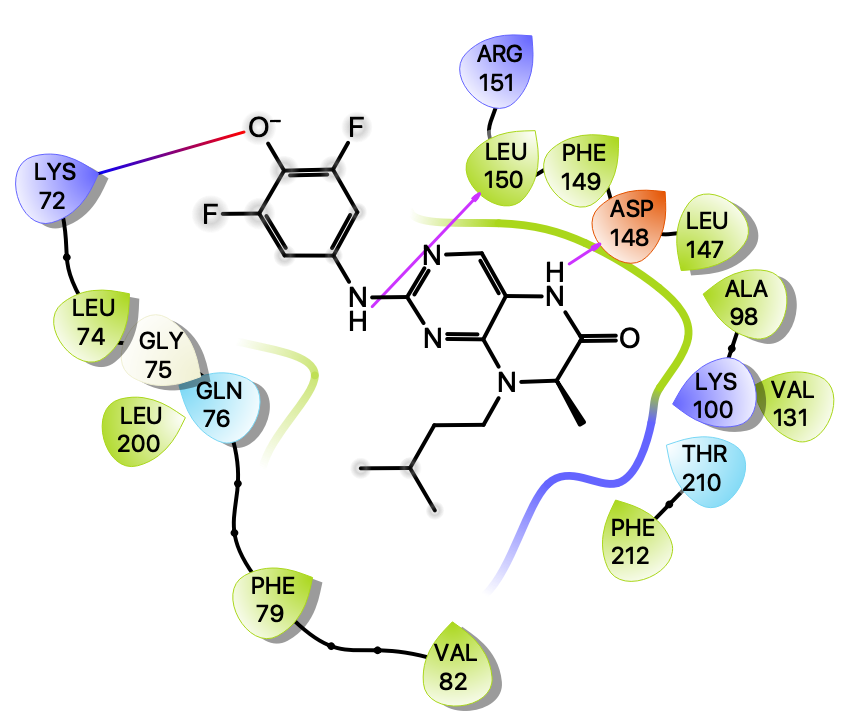

Supplement: Multimedia component 1 [file mmc1.zip › Schrodinger/Ligand Interaction Maps/26.png]

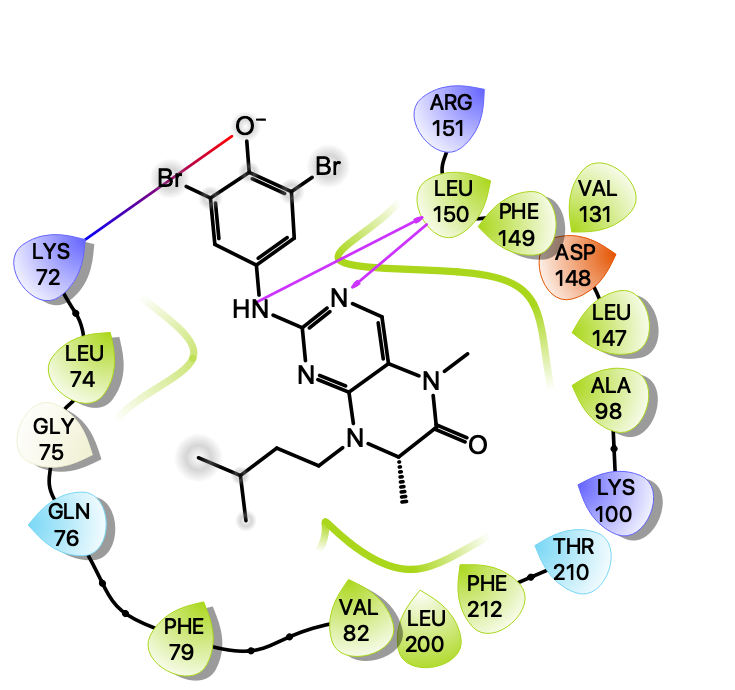

Supplement: Multimedia component 1 [file mmc1.zip › Schrodinger/Ligand Interaction Maps/27.png]

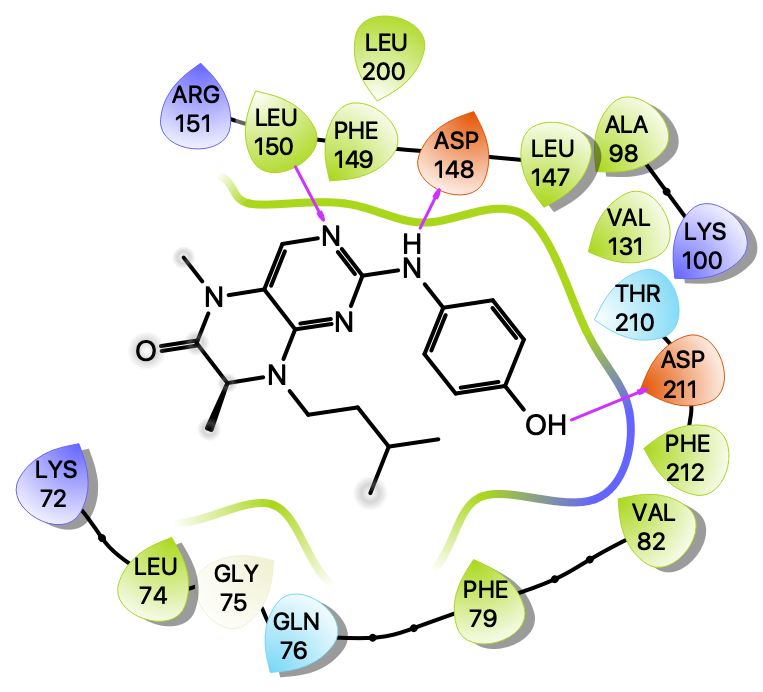

Supplement: Multimedia component 1 [file mmc1.zip › Schrodinger/Ligand Interaction Maps/28.png]

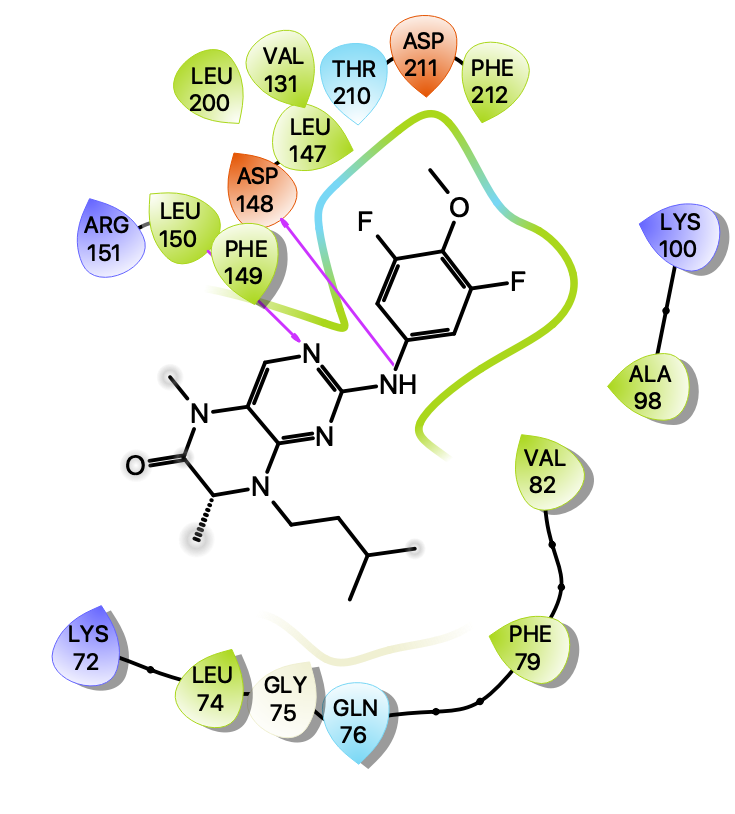

Supplement: Multimedia component 1 [file mmc1.zip › Schrodinger/Ligand Interaction Maps/29.png]

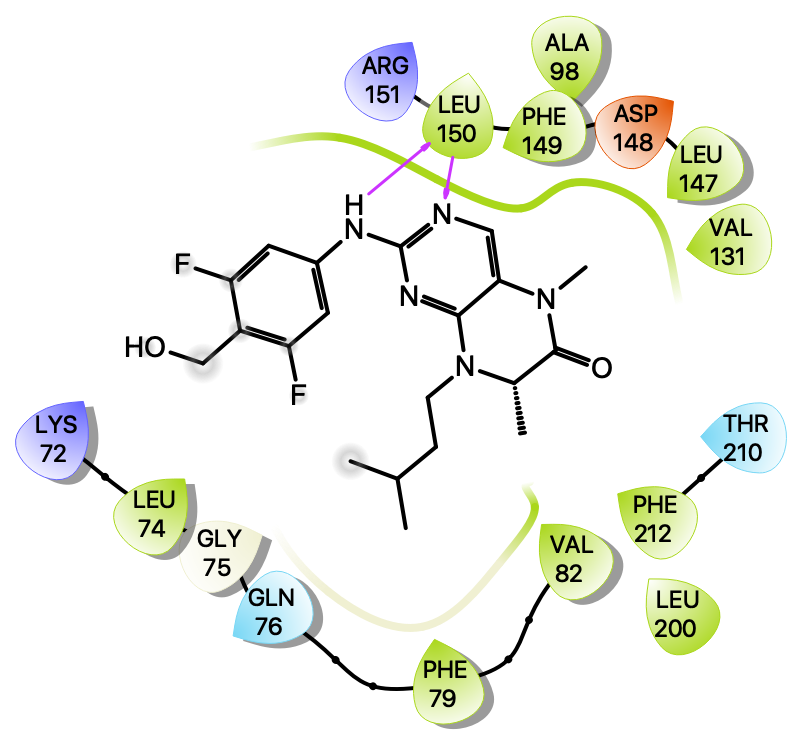

Supplement: Multimedia component 1 [file mmc1.zip › Schrodinger/Ligand Interaction Maps/30.png]

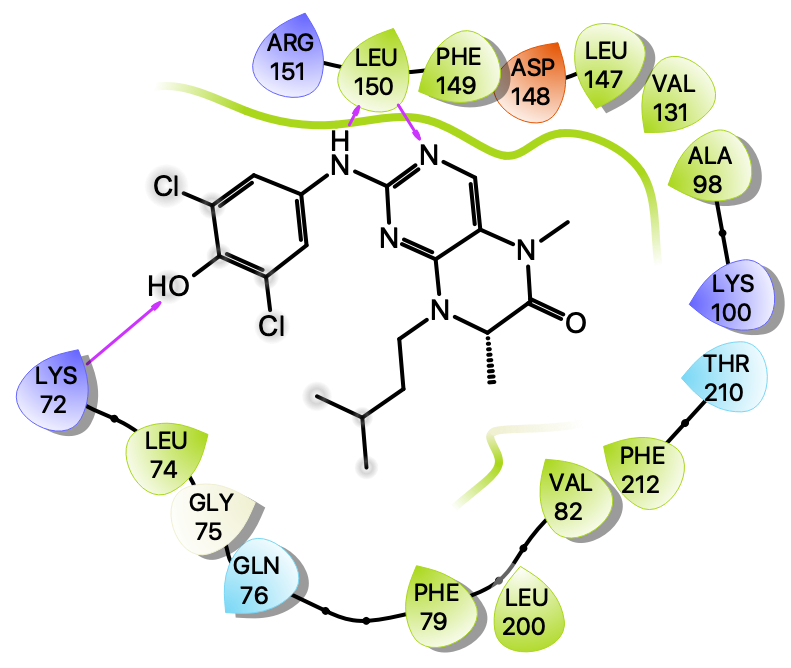

Supplement: Multimedia component 1 [file mmc1.zip › Schrodinger/Ligand Interaction Maps/31.png]

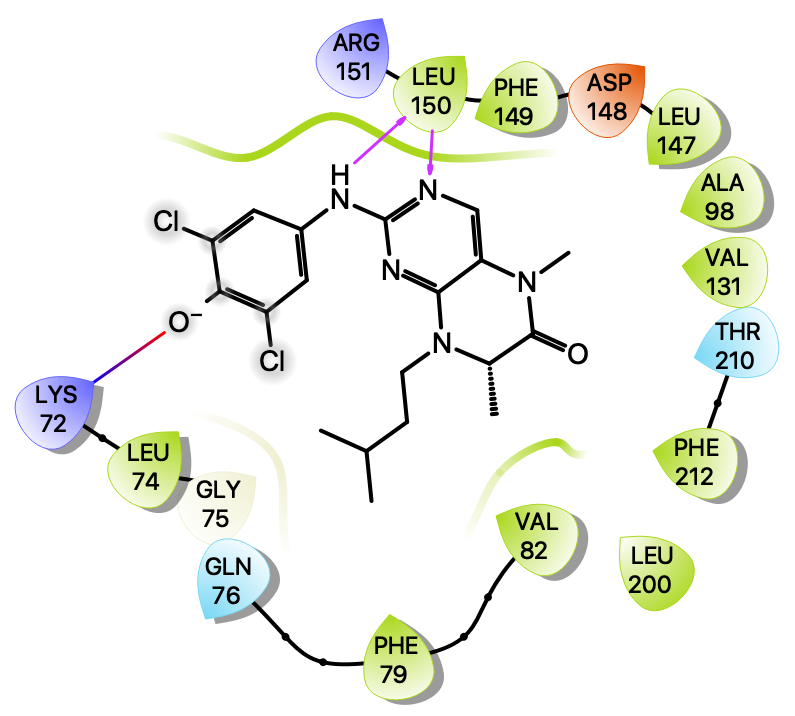

Supplement: Multimedia component 1 [file mmc1.zip › Schrodinger/Ligand Interaction Maps/32.png]

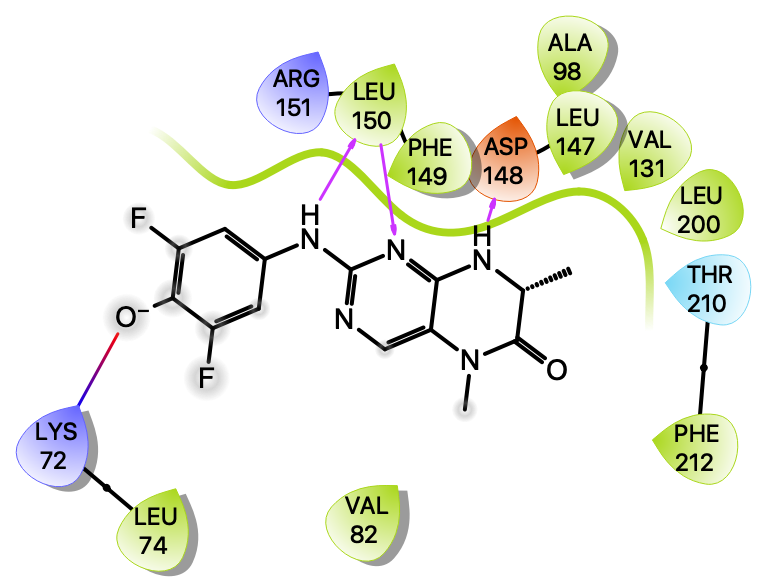

Supplement: Multimedia component 1 [file mmc1.zip › Schrodinger/Ligand Interaction Maps/33.png]

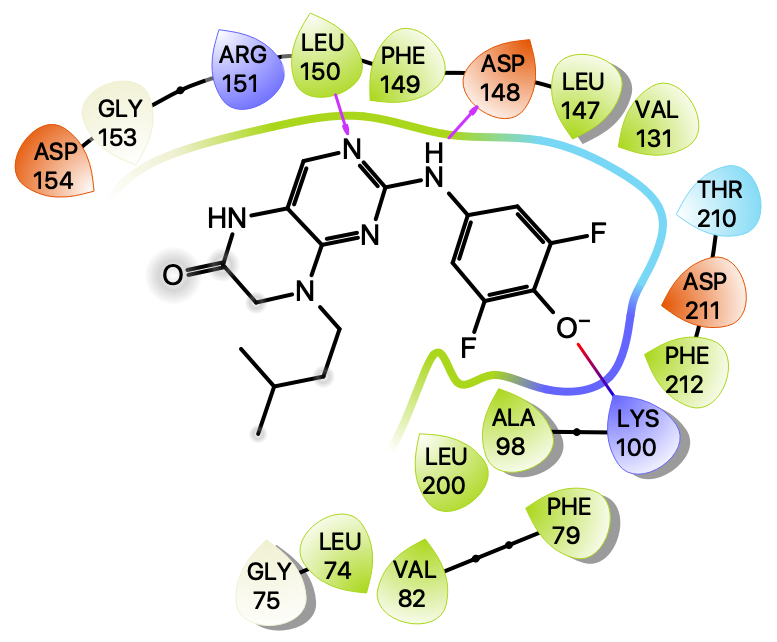

Supplement: Multimedia component 1 [file mmc1.zip › Schrodinger/Ligand Interaction Maps/34.png]

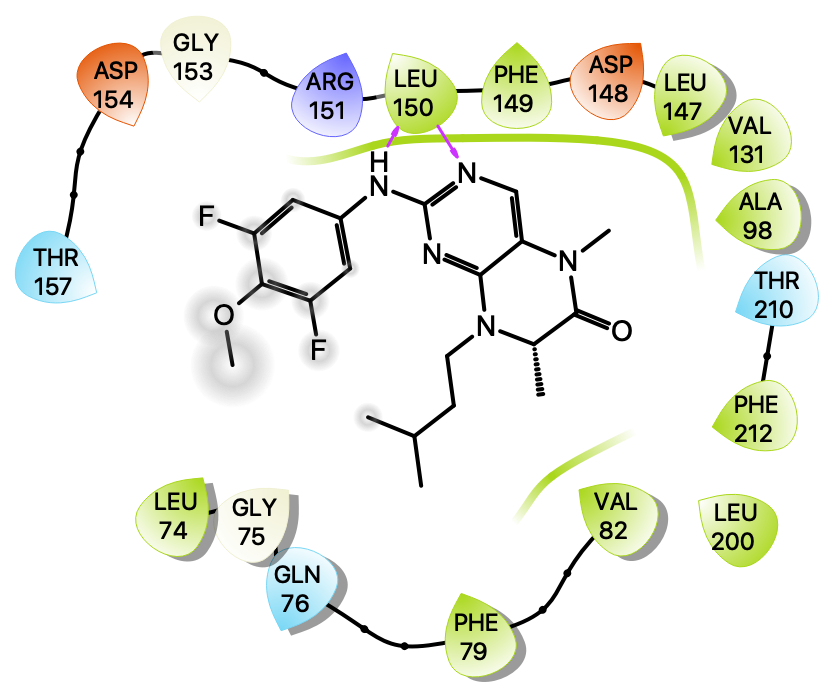

Supplement: Multimedia component 1 [file mmc1.zip › Schrodinger/Ligand Interaction Maps/35.png]

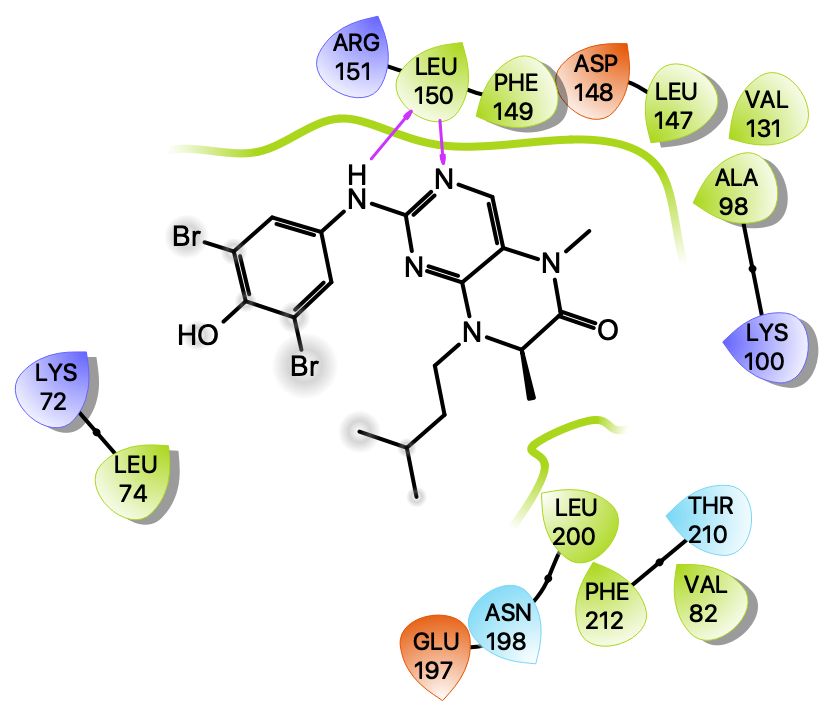

Supplement: Multimedia component 1 [file mmc1.zip › Schrodinger/Ligand Interaction Maps/36.png]

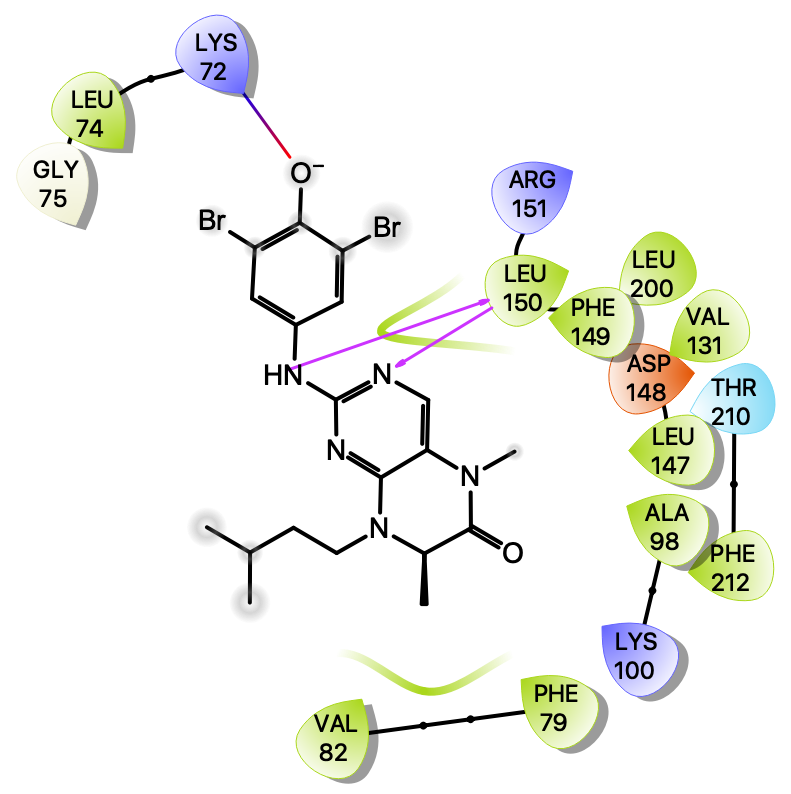

Supplement: Multimedia component 1 [file mmc1.zip › Schrodinger/Ligand Interaction Maps/37.png]

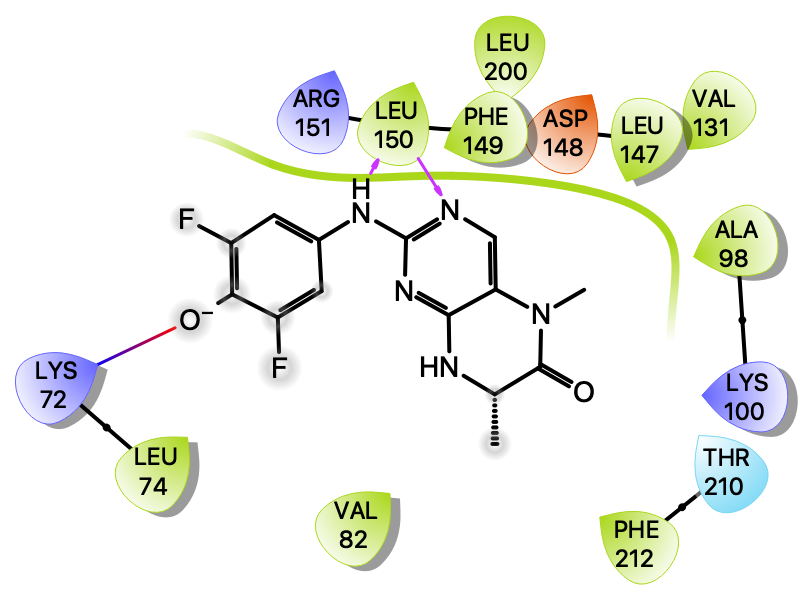

Supplement: Multimedia component 1 [file mmc1.zip › Schrodinger/Ligand Interaction Maps/38.png]

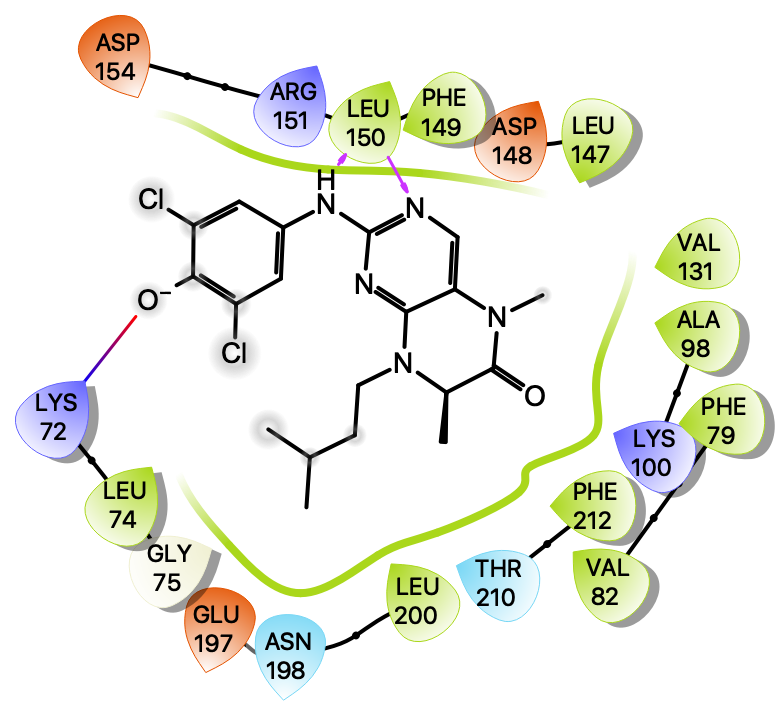

Supplement: Multimedia component 1 [file mmc1.zip › Schrodinger/Ligand Interaction Maps/39.png]

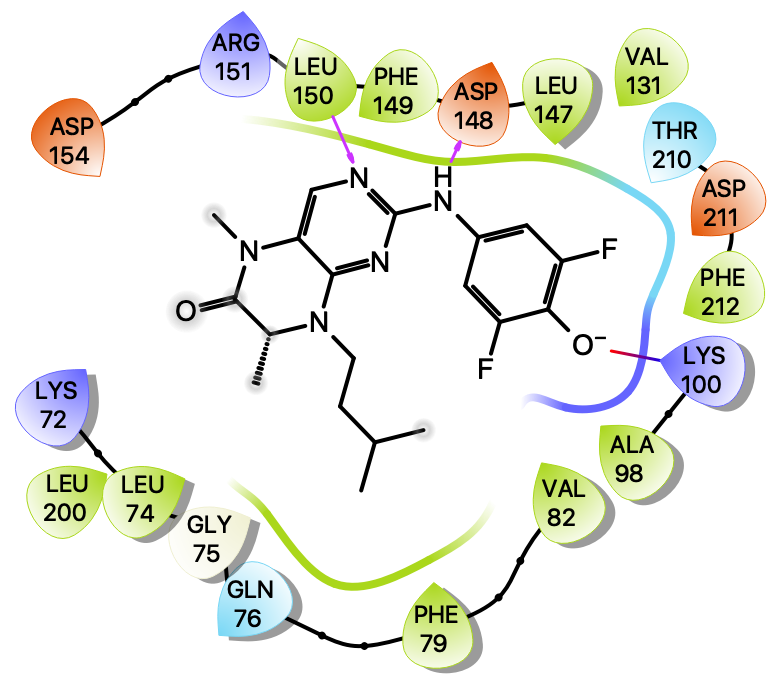

Supplement: Multimedia component 1 [file mmc1.zip › Schrodinger/Ligand Interaction Maps/40.png]

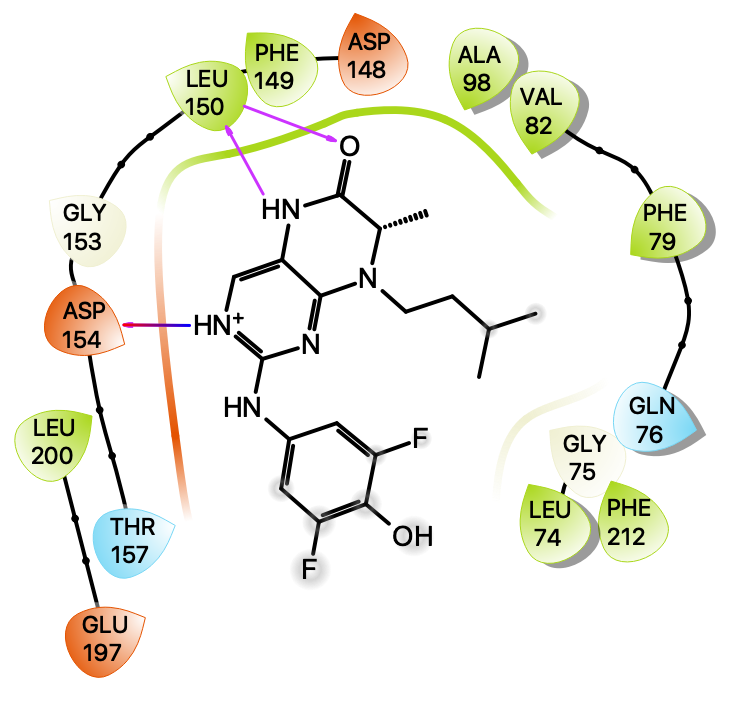

Supplement: Multimedia component 1 [file mmc1.zip › Schrodinger/Ligand Interaction Maps/41.png]

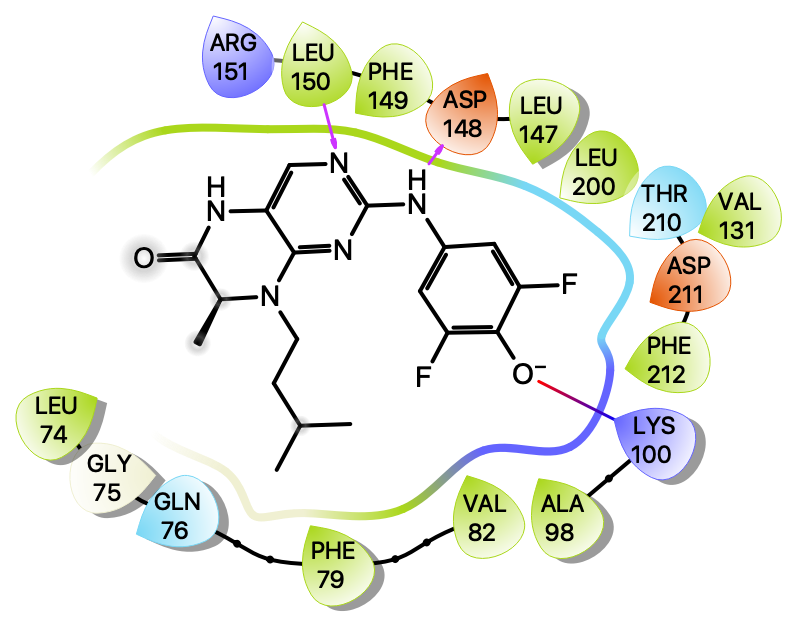

Supplement: Multimedia component 1 [file mmc1.zip › Schrodinger/Ligand Interaction Maps/42.png]

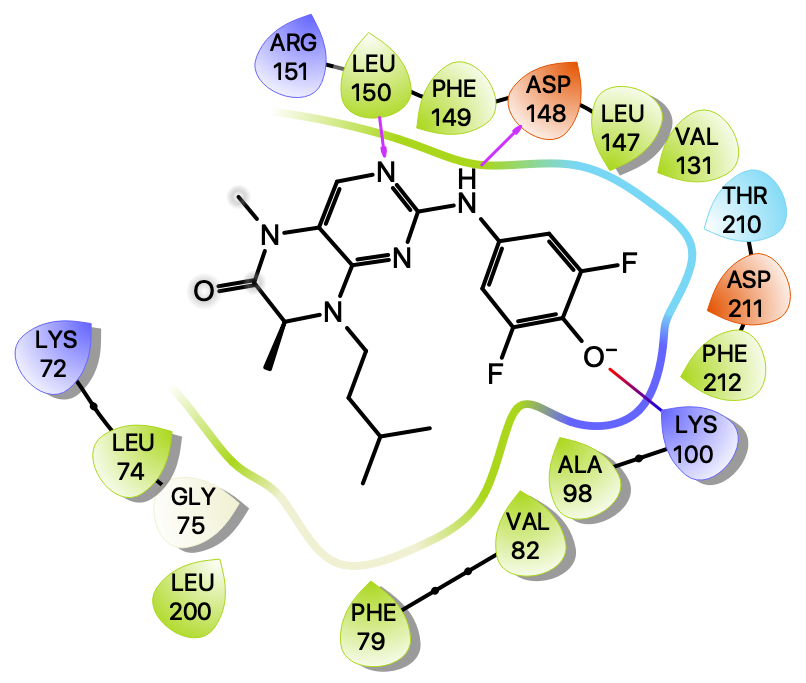

Supplement: Multimedia component 1 [file mmc1.zip › Schrodinger/Ligand Interaction Maps/43.png]

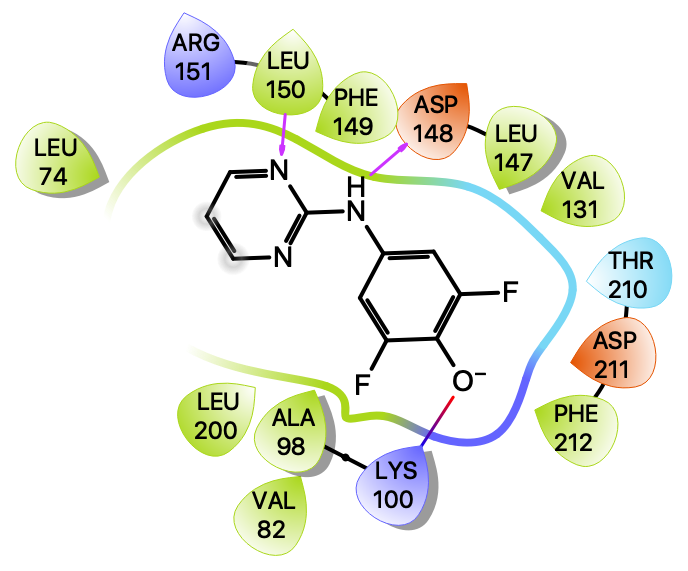

Supplement: Multimedia component 1 [file mmc1.zip › Schrodinger/Ligand Interaction Maps/44.png]

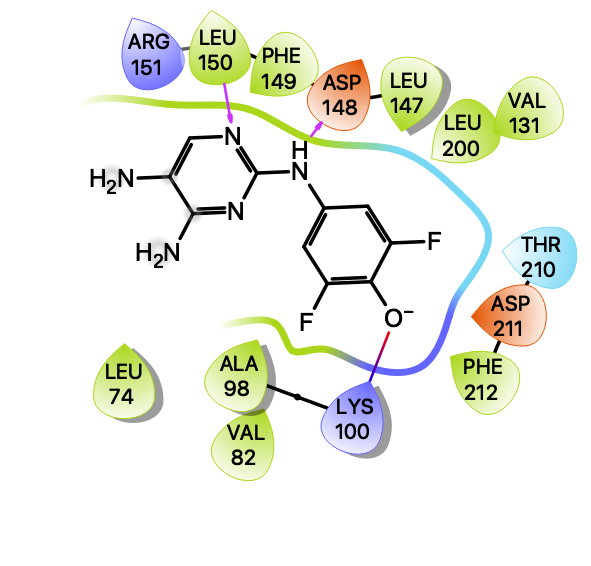

Supplement: Multimedia component 1 [file mmc1.zip › Schrodinger/Ligand Interaction Maps/45.png]

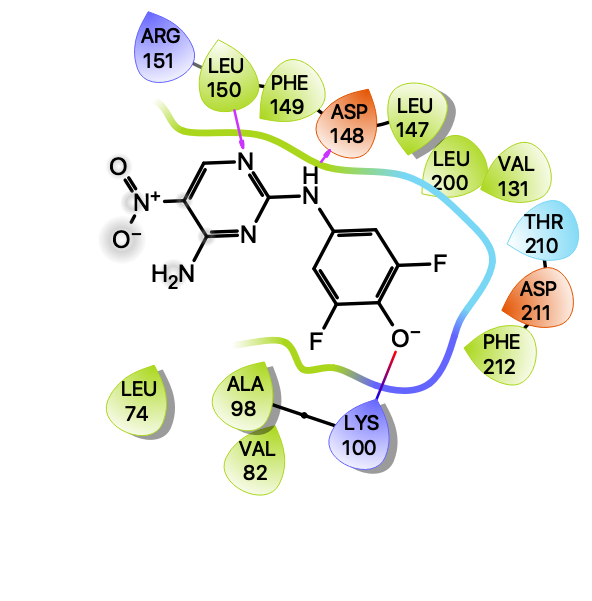

Supplement: Multimedia component 1 [file mmc1.zip › Schrodinger/Ligand Interaction Maps/46.png]

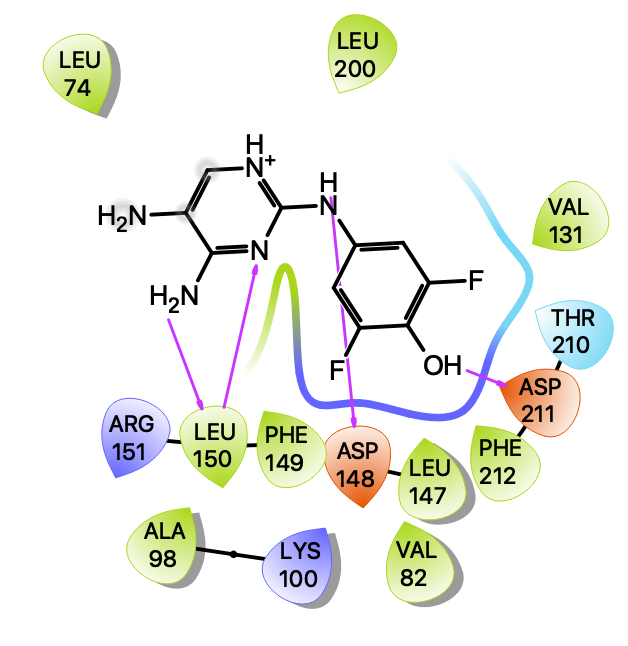

Supplement: Multimedia component 1 [file mmc1.zip › Schrodinger/Ligand Interaction Maps/47.png]

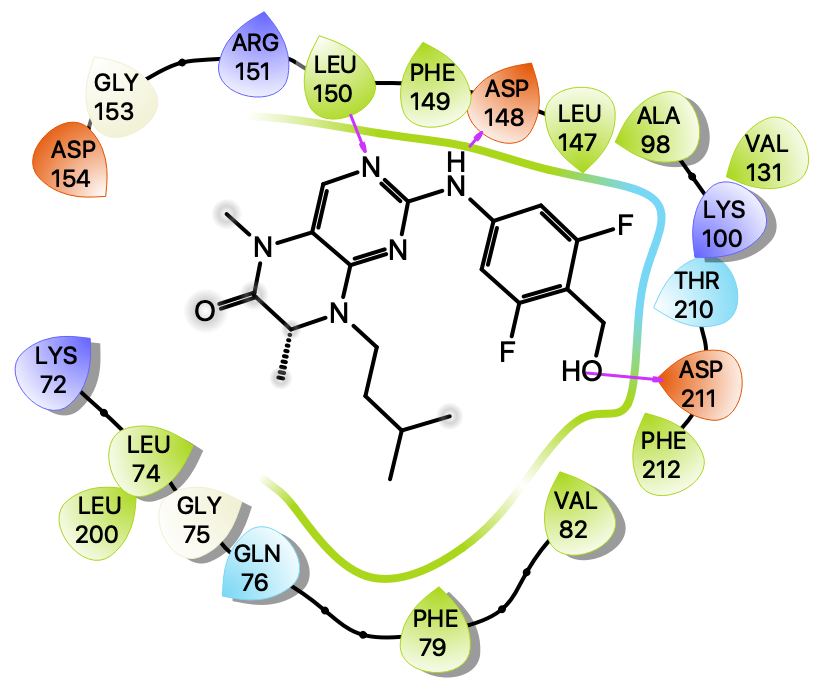

Supplement: Multimedia component 1 [file mmc1.zip › Schrodinger/Ligand Interaction Maps/48.png]

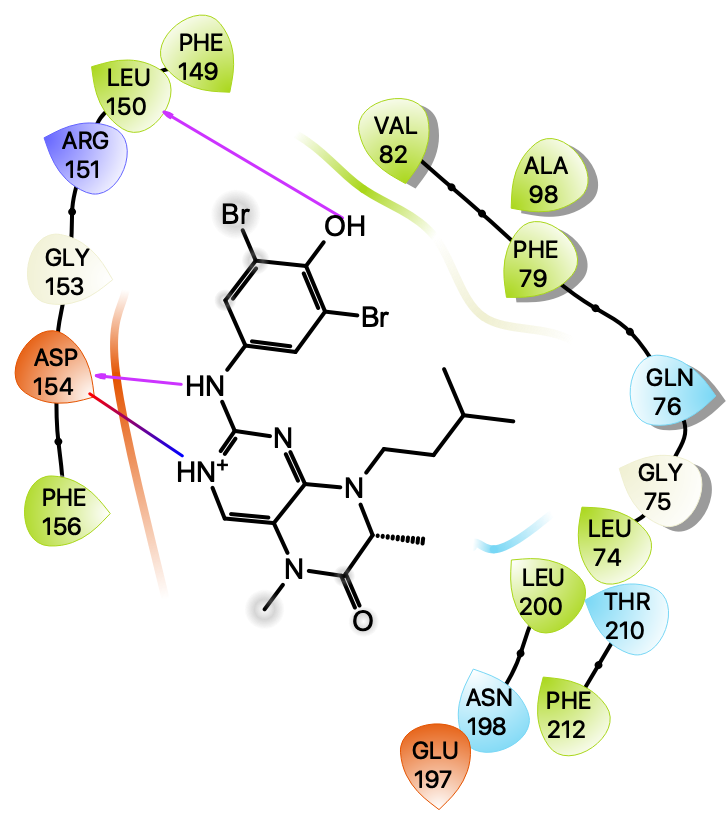

Supplement: Multimedia component 1 [file mmc1.zip › Schrodinger/Ligand Interaction Maps/49.png]

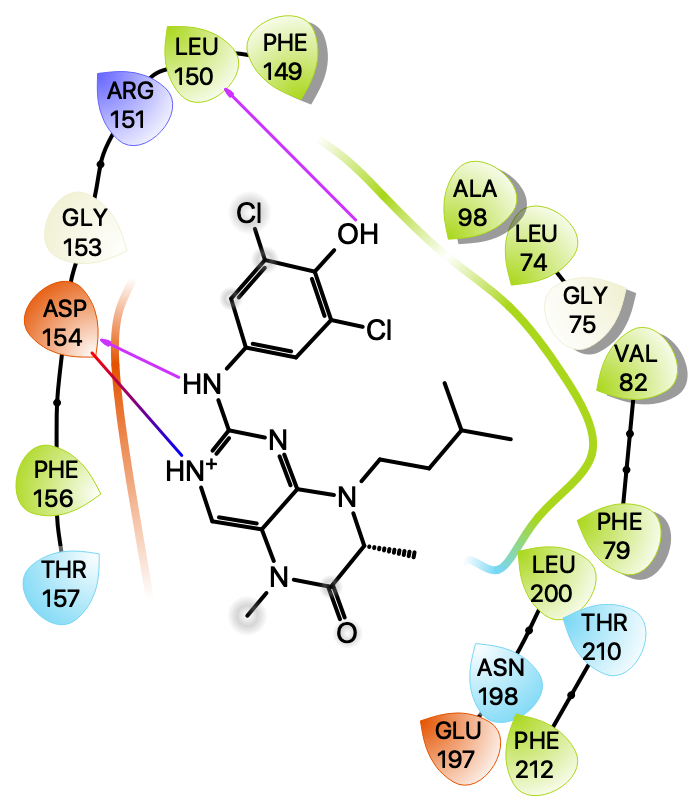

Supplement: Multimedia component 1 [file mmc1.zip › Schrodinger/Ligand Interaction Maps/50.png]

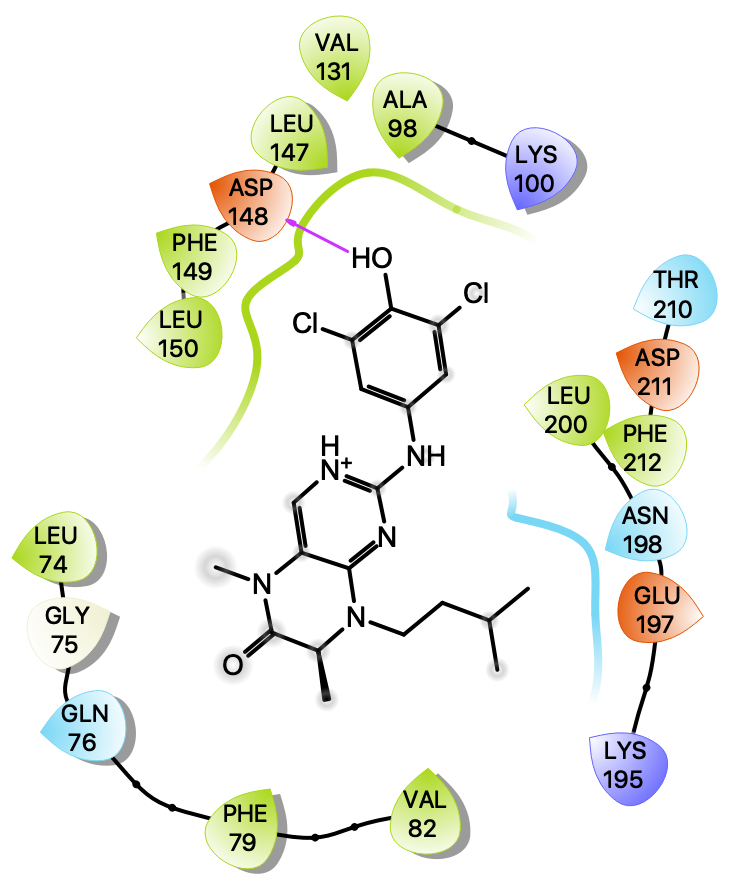

Supplement: Multimedia component 1 [file mmc1.zip › Schrodinger/Ligand Interaction Maps/51.png]

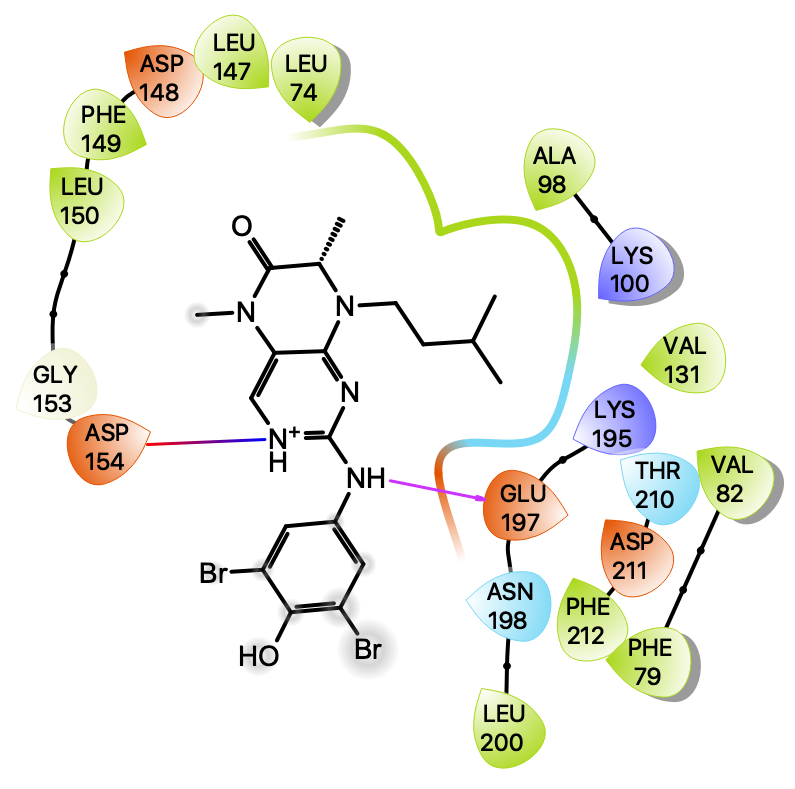

Supplement: Multimedia component 1 [file mmc1.zip › Schrodinger/Ligand Interaction Maps/52.png]

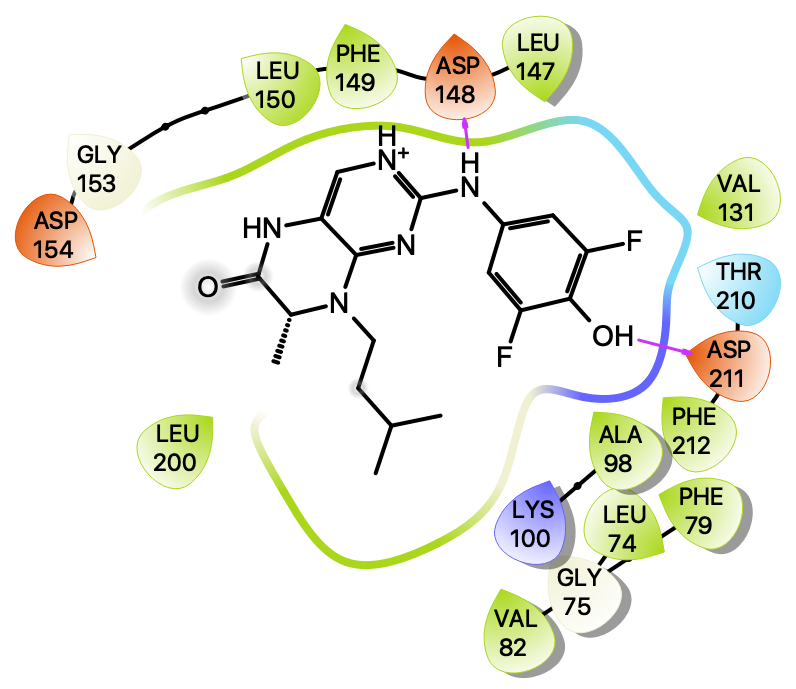

Supplement: Multimedia component 1 [file mmc1.zip › Schrodinger/Ligand Interaction Maps/53.png]

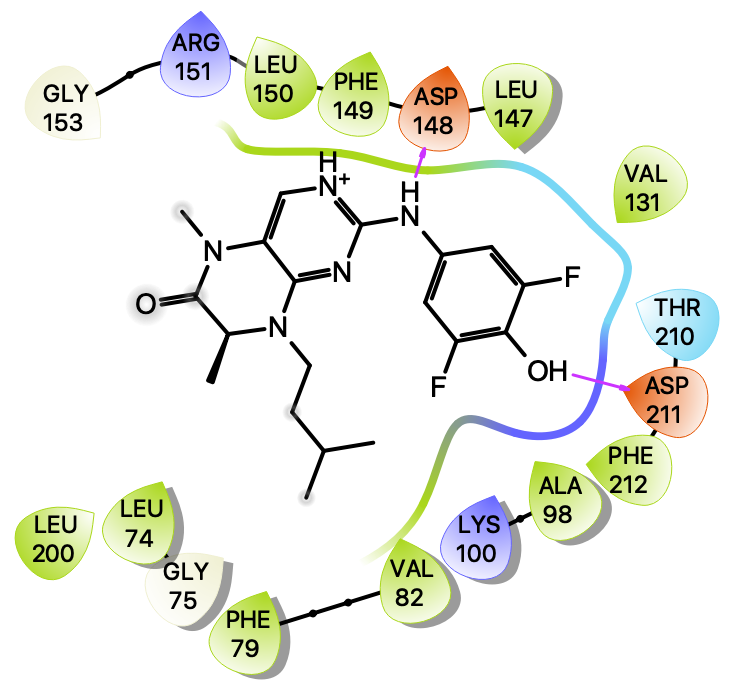

Supplement: Multimedia component 1 [file mmc1.zip › Schrodinger/Ligand Interaction Maps/54.png]

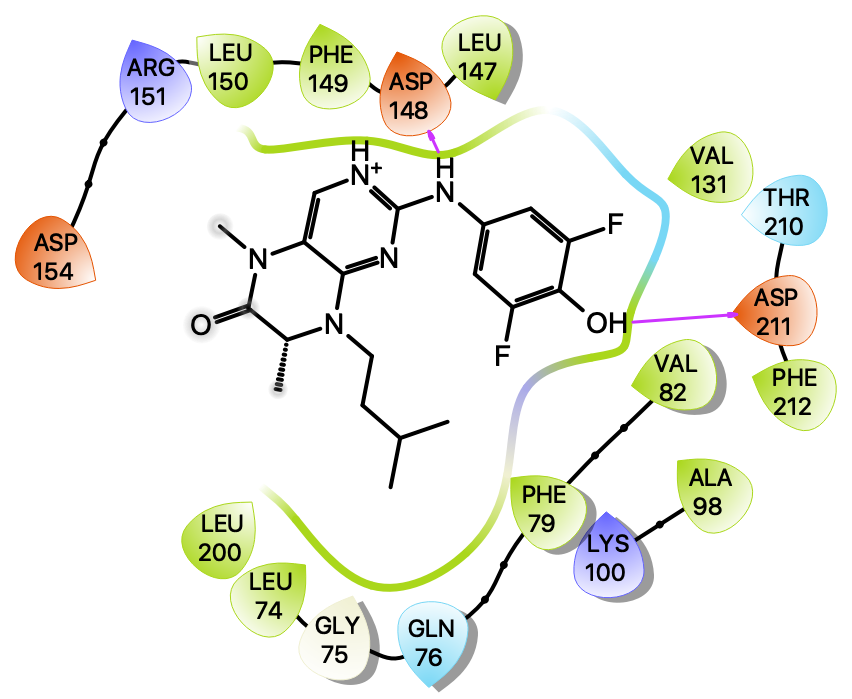

Supplement: Multimedia component 1 [file mmc1.zip › Schrodinger/Ligand Interaction Maps/55.png]

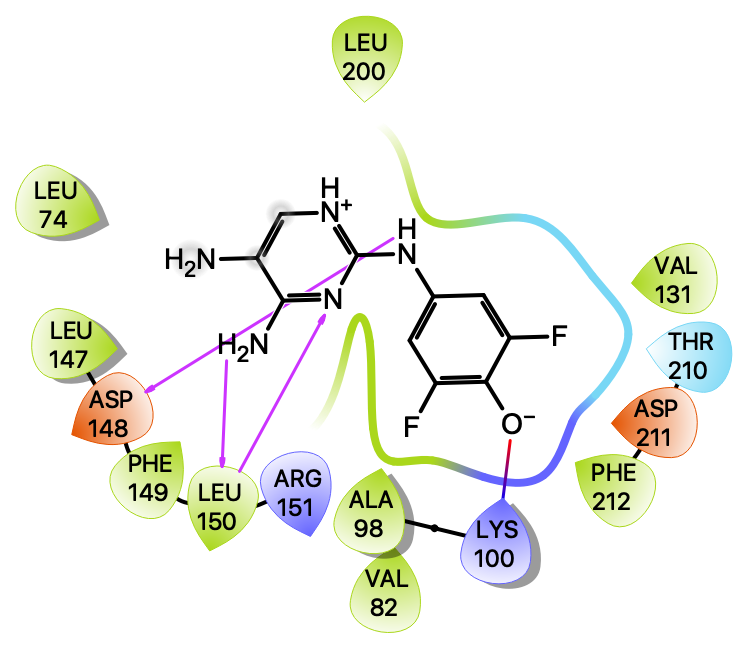

Supplement: Multimedia component 1 [file mmc1.zip › Schrodinger/Ligand Interaction Maps/56.png]

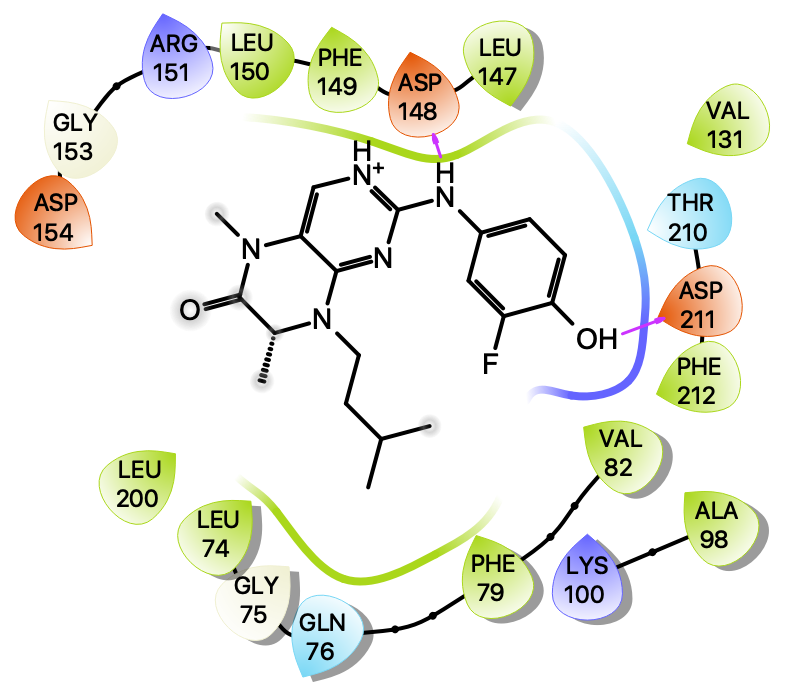

Supplement: Multimedia component 1 [file mmc1.zip › Schrodinger/Ligand Interaction Maps/57.png]

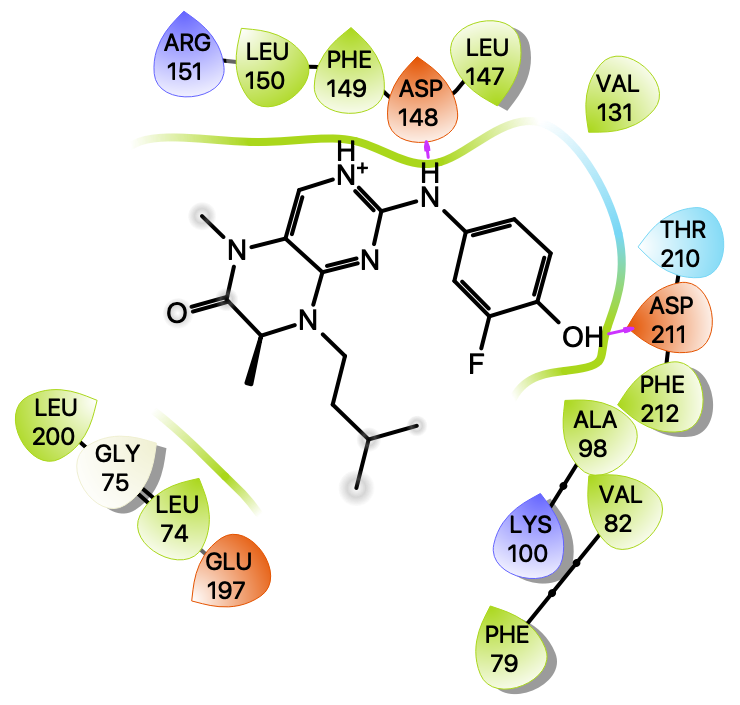

Supplement: Multimedia component 1 [file mmc1.zip › Schrodinger/Ligand Interaction Maps/58.png]

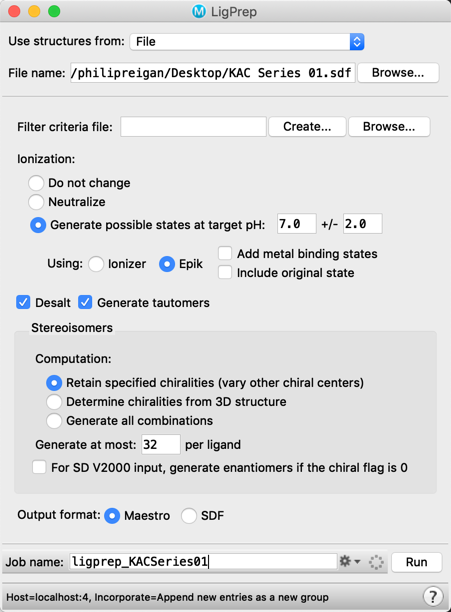

Supplement: Multimedia component 1 [file mmc1.zip › Schrodinger/Parameters/2019-12-09_16-41-43.png]

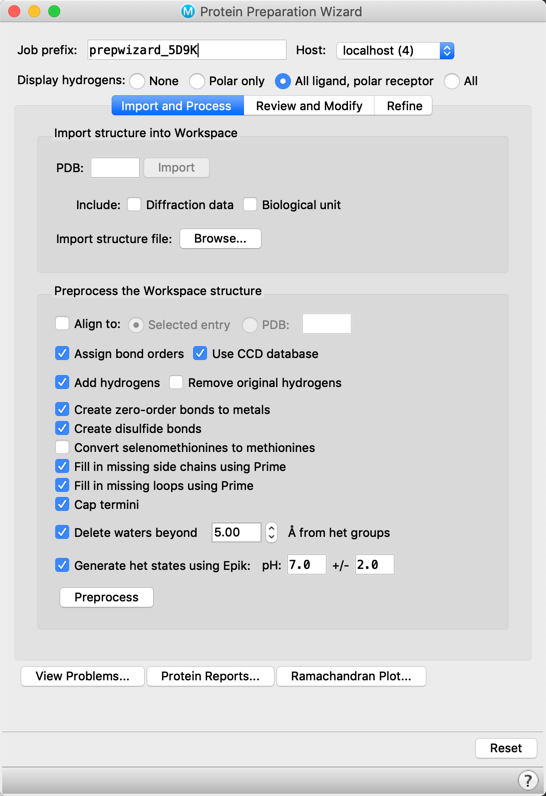

Supplement: Multimedia component 1 [file mmc1.zip › Schrodinger/Parameters/2019-12-09_16-42-49.png]

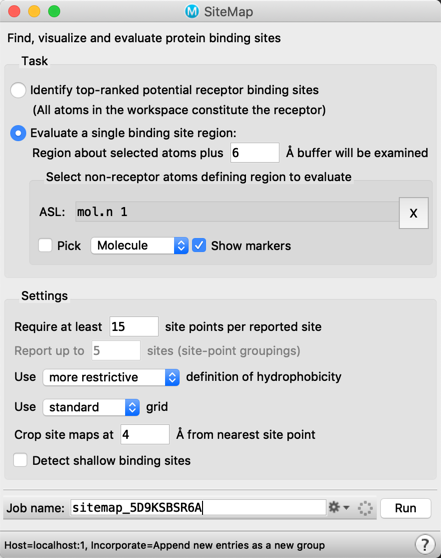

Supplement: Multimedia component 1 [file mmc1.zip › Schrodinger/Parameters/2019-12-09_16-48-02.png]

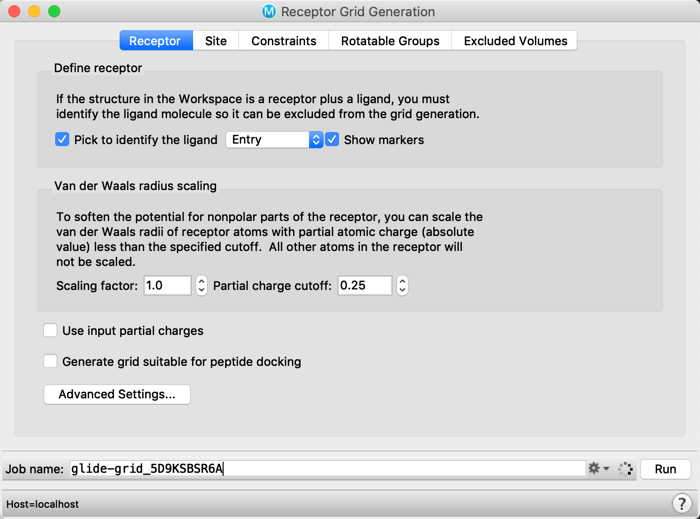

Supplement: Multimedia component 1 [file mmc1.zip › Schrodinger/Parameters/2019-12-09_17-02-22.png]

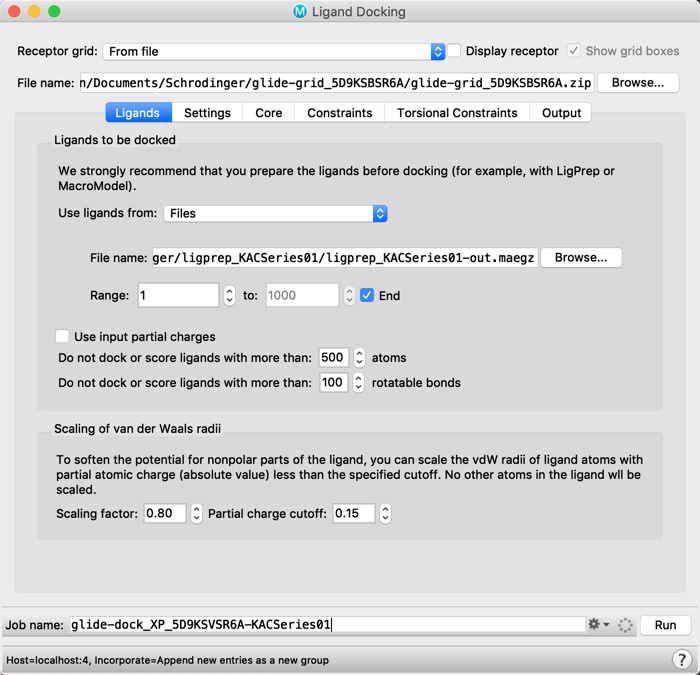

Supplement: Multimedia component 1 [file mmc1.zip › Schrodinger/Parameters/2019-12-09_17-31-05.png]

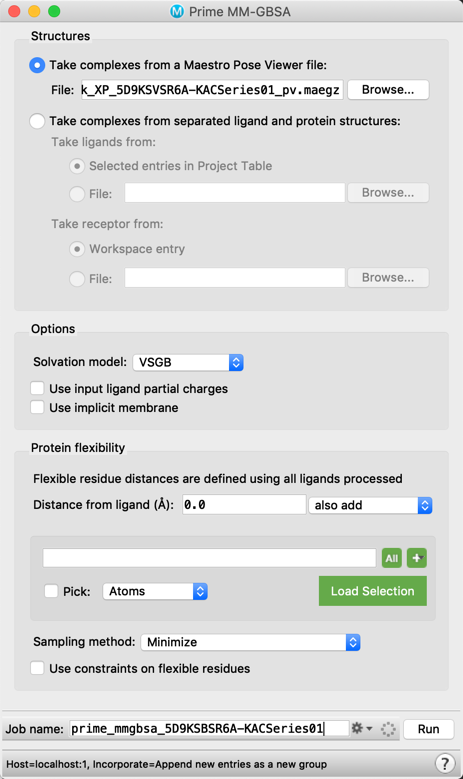

Supplement: Multimedia component 1 [file mmc1.zip › Schrodinger/Parameters/2019-12-09_19-11-53.png]
